# Vegan versus meat-based dog food: Guardian-reported indicators of health

**DOI:** 10.1371/journal.pone.0265662

**Published:** 2022-04-13

**Authors:** Andrew Knight, Eason Huang, Nicholas Rai, Hazel Brown

**Affiliations:** 1 Centre for Animal Welfare, Faculty of Health and Wellbeing, University of Winchester, Winchester, United Kingdom; 2 School of Environment and Science, Nathan Campus, Griffith University, Nathan, QLD, Australia; 3 Independent consultant, Rochedale, Brisbane, QLD, Australia; 4 Menzies Health Institute Queensland, Griffith University, Nathan, QLD, Australia; University of Lincoln, UNITED KINGDOM

## Abstract

Alternative pet foods may offer benefits concerning environmental sustainability and the welfare of animals processed into pet foods. However, some worry these may compromise the welfare of pets. We asked 2,639 dog guardians about one dog living with them, for at least one year. Among 2,596 involved in pet diet decision-making, pet health was a key factor when choosing diets. 2,536 provided information relating to a single dog, fed a conventional meat (1,370 = 54%), raw meat (830 = 33%) or vegan (336 = 13%) diet for at least one year. We examined seven general indicators of ill health: unusual numbers of veterinary visits, medication use, progression onto a therapeutic diet after initial maintenance on a vegan or meat-based diet, guardian opinion and predicted veterinary opinion of health status, percentage of unwell dogs and number of health disorders per unwell dog. Dogs fed conventional diets appeared to fare worse than those fed either of the other two diets. Dogs fed raw meat appeared to fare marginally better than those fed vegan diets. However, there were statistically significant differences in average ages. Dogs fed raw meat were younger, which has been demonstrated to be associated with improved health outcomes. Additionally, non-health related factors may have improved apparent outcomes for dogs fed raw meat, for three of seven general health indicators. We also considered the prevalence of 22 specific health disorders, based on predicted veterinary assessments. Percentages of dogs in each dietary group considered to have suffered from health disorders were 49% (conventional meat), 43% (raw meat) and 36% (vegan). Significant evidence indicates that raw meat diets are often associated with dietary hazards, including nutritional deficiencies and imbalances, and pathogens. Accordingly, the pooled evidence to date indicates that the healthiest and least hazardous dietary choices for dogs, are nutritionally sound vegan diets.

## Introduction

In 2018, the global pet population was estimated to include 471 million dogs, and 373 million cats [1] (p. 4). Pet food sales internationally were worth Euro 131.7 billion in 2014 [2]. The UK pet food market was expected to reach GBP 2.8 billion by the end of 2019, having risen 17% over the previous five years [3], and US pet food and treat sales were also rising, being valued at USD 42.0 billion by 2020 [4].

A market of such size drives considerable research and product development, and between January 2013 and October 2014, over 6,000 new petfood products (3,000 dry and 3,200 wet pet foods), as well as 4,000 new pet snacks, were launched globally [5] (in [6]). Some of the new products being developed include raw meat diets, *in vitro* meat products, and diets based on novel protein sources, including terrestrial plants, insects, yeast, fungi and seaweed. Some of this development may be driven by significant recent concerns about the environmental sustainability of animal agriculture, and of traditional pet foods based on animal produce [7–11]. This market is already large, and is growing fast. The vegan pet food market was worth USD 8.7 billion globally by 2020, and its forecast valuation by 2028 has been estimated at USD 15.7 billion–a compound annual growth rate of 7.7% [12].

However, concerns exist that the imposition of human petfood preferences may be suboptimal for the welfare of dogs. Claiming the existence of “almost insurmountable challenges–biological, legal and downright practical–facing anyone attempting to shoehorn dogs and cats into a vegan dietary system”, Loeb [13] stated, “it’s fairly clear that feeding a dog a vegan diet is not recommended.”

How valid are such concerns about the nutritional suitability for dogs, of vegan diets? There are two clear routes to assess this. The first involves examining steps taken by petfood manufacturers to ensure the quality and nutritional soundness of their products. These were recently examined in a survey of 29 companies producing meat-based (19) and plant-based (10) pet foods [14]. Although there were limited areas in which practices could be improved, most manufacturers had acceptable or superior standards at nearly all stages examined, throughout the design, manufacturing, transportation and storage phases, with plant-based diets slightly superior to meat-based diets overall.

However, the most important test is always the effect(s), if any, on the animals themselves. This is why feeding trials are considered the gold standard to ensure the nutritional soundness of new formulations [15, 16]. The health status of dogs maintained on different diets has been the subject of limited studies to date, some of which we’ve reviewed elsewhere [17]. In 1987, Yamada and colleagues [18] reported the results of a study of eight dogs divided into two groups, maintained on commercial animal or vegetable protein (VP)-based diets. Each comprised around 30% protein, and other macronutrients and energy contents were closely matched. Six weeks of rest was followed by four hours daily of enforced running at 12 km/h for two weeks. This was followed by one week of recovery. Blood tests indicated that the VP-based dogs experienced marked anaemia following this relatively severe exercise regime. This was theorised to be due to changes in circulating lipid levels (reduction of free cholesterol), resulting in lowered erythrocyte resistance to haemolysis.

In 2009, Brown and colleagues [19] reported conflicting results, from a marginally larger study of 12 sprint-racing Siberian Huskies, fed either a commercial meat-based diet recommended for active dogs (n = 6), or a meat free (although not vegan) diet formulated to the same nutrient specifications (n = 6). The dogs were fed these diets for 16 weeks, including 10 weeks of competitive racing. Blood tests were conducted on four occasions, and veterinary health checks on three occasions. All dogs were assessed as being in excellent physical condition, and none developed anaemia or other detectable health problems.

Additionally, in 2014 Semp [20] reported a study of vegan companion animals in Austria, Germany and Switzerland. A questionnaire was completed by 174 dog and 59 cat guardians, some of whom had both species. Participating dogs had eaten vegan diets for six months to seven years, with a mean of 2.83 years. Clinical examinations and blood tests were conducted on 20 randomly selected dogs. No diet-related clinical abnormalities were detected. Haematological (complete blood count) and biochemical (liver, kidney, and pancreatic) parameters were assessed, as well as levels of magnesium, calcium, iron, total protein, folic acid, vitamin B12, and carnitine. The serum total protein of all dogs was within normal ranges. No significant differences were evident in any tested parameters, compared to dogs fed a conventional diet. Not even the 10% (2/20) of dogs fed a homemade supplemented diet showed any significant deviations.

However, the relatively small numbers included in these samples limits their predictive value for wider dog populations. By 2021, no large-scale study of dogs had been published, describing how health indicators vary between dogs maintained on vegan, meat-based, or indeed, other diets. Accordingly, we designed a survey to explore this. Our null hypothesis was that guardian-reported canine health indicators would not significantly vary with diet type. The success of new pet foods under development also depends on the views of consumers. We also sought to determine the importance of pet health as a purchasing determinant, to a large group of dog guardians. Results of some survey parts were recently reported (palatability of different diets; [21]), or are the subject of related, forthcoming studies.

## Methodology

We designed a survey for dog or cat guardians using the ‘Online surveys’ platform (https://www.onlinesurveys.ac.uk). Guardians were asked to provide information about themselves and one dog or cat resident within their household for at least one year. Guardians were asked about the main ingredients within their pet’s normal diet. They were asked to identify whether the diet was based on conventional, raw or *in vitro* meat, insects, fungi or algae, or whether it was a vegetarian, vegan or some ‘other’ diet. Respondents could select only one option. Vegetarian diets were explained as including eggs or milk, but not meat, and vegan diets as eschewing any animal products. Where animals were fed a prescription or therapeutic diet, guardians were asked to base answers on the diet in use prior to the commencement of the therapeutic diet. Guardians were also asked about any treats/snacks/scraps or supplements provided.

Our survey also inquired about human demographics (continental region, urban or rural location, educational qualifications achieved, occupation, household income, age categories in 10 year age bands with the exception of bands for 18–19 and ‘70 or older’, gender, and respondent diet). Information was also obtained about animal demographics. These included role (companion or working animal), age (with any year entry up to ‘over 25’ possible), sex/neuter status, canine breed (toy, small, medium, large and giant), activity level, health status, reaction to meals, factors of importance to guardians when choosing pet food, and information sources guardians relied on.

Guardians provided information about seven general indicators of health, and about the prevalence of specific health disorders, for the previous year, or the year prior to the commencement of a therapeutic diet if one was currently used. Specifically, guardians were asked to report the frequency of veterinary visits, and medication use (other than routine vaccinations and treatments for external or internal parasites, such as fleas, ticks, lice, heartworm and intestinal worms, or treatments associated with neutering operations, or microchipping). Guardians were asked to report whether their dog had progressed onto a therapeutic diet, after initial maintenance on another diet type. They were asked to report their own opinion of their dog’s health status, and also to report what they believed their veterinarian’s assessment to be. Guardians were asked to “Think about your veterinarian. Which of the following would most likely describe their opinions about your animal’s medical condition over the previous 12 months?” Possible answers for both their own opinions, and for the reported assessments of their veterinarians, ranged from no problems/routine preventative healthcare, to seriously ill. If veterinarians reportedly considered dogs to be suffering from health disorder(s), guardians were asked which disorder(s) these were, from among 18 disorders indicated to be among the most common disorders experienced by companion dogs [22–26]. Guardians were able to select multiple disorders, and to provide details of additional disorders by selecting ‘other’. Details for each ‘other’ entry were examined, with these entries then reclassified into 18 existing or four new disorder types, giving a total of 22 possible health disorders.

When analysing health disorders, cases were excluded, where veterinary visits had not occurred at least once in the previous year, or where guardians were unsure of the assessments of their veterinarians. The remaining subset comprised guardians who had recently seen their veterinarians, and were sure of their health assessments. This subset was used to calculate the percentage of unwell dogs, and the average number of cases of disorder, per dog. It was also used to calculate the prevalence of the 22 specific health disorders.

### Potentially confounding factors

Health status may be affected by age, sex, desexing (neutering) status and breed [25–28]. Hence, we sought to ascertain differences between major dietary groups, in age, sex and neuter status. We decided not to attempt to account for the possible effects of certain additional factors, on health outcomes. Breed, for example, can affect health status [27, 28]. However, we were concerned that small numbers within breed groups would limit our ability to statistically analyse subsequent results, and so ultimately elected not to discriminate by breed within this study. We also chose not to exclude dogs who received treats regularly, expecting most to receive such treats.

### Survey pilot and distribution

Our survey piloting and distribution were described in Knight and Satchell [21]. The ‘Online surveys’ platform we chose to use complies with the UK General Data Protection Regulation, following the UK Data Protection Act 2018, and was used by 88% of UK higher education institutions by 2019 [29], including our University of Winchester.

**Fig 1:**
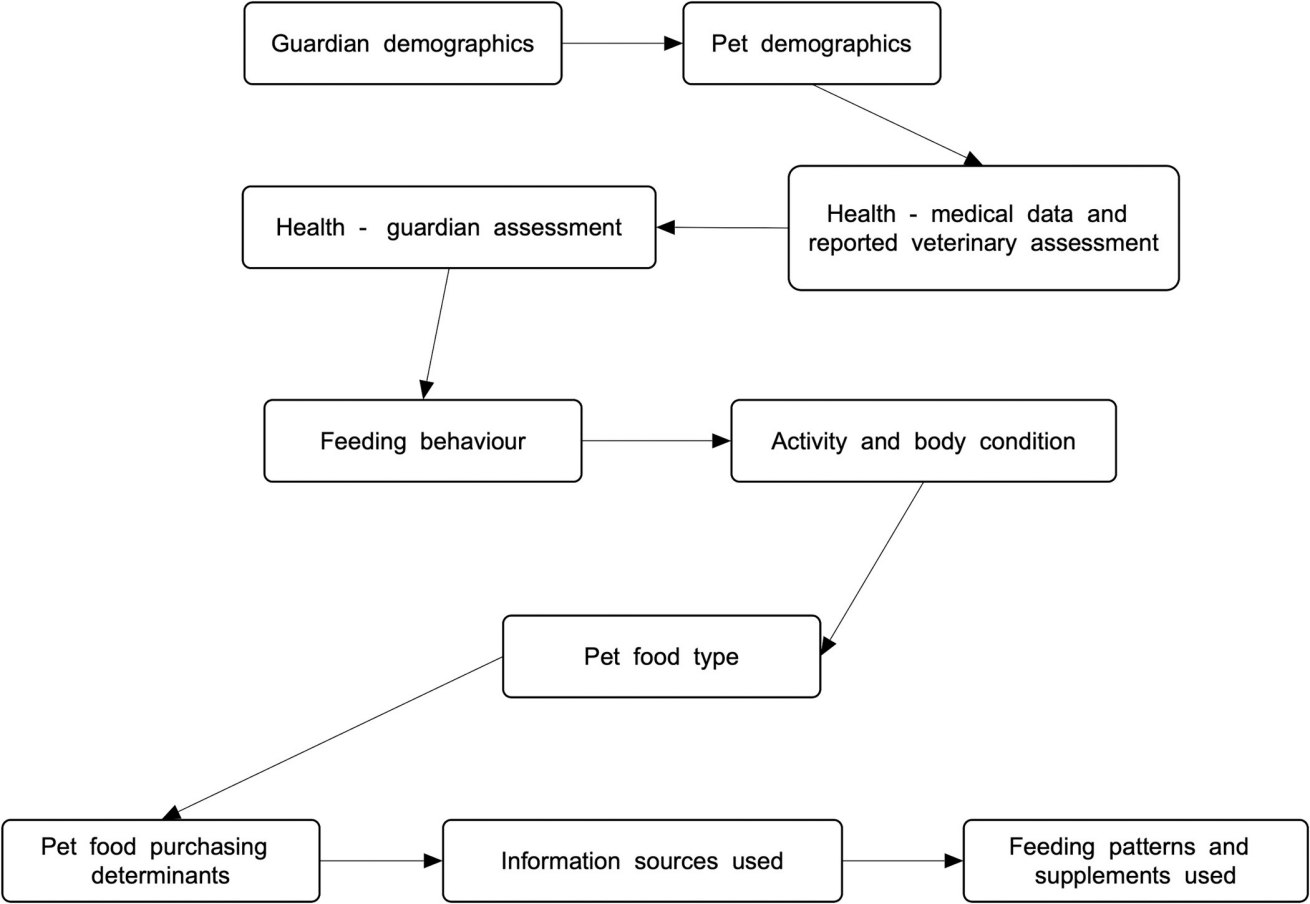
Survey parts.

We piloted our survey to 25 respondents in April 2020. Improvements were then made to both survey structure and questions. With respect to structure, changes were made to the ordering of survey parts, to minimise inadvertently biasing answers to questions about health. These survey sections were moved toward the beginning, to eliminate chances that answers might be affected by prior answers about pet diet choices, particularly where unconventional diets were used, e.g., if a guardian reporting use of an unconventional diet might subsequently be more likely to consciously or unconsciously downplay any health problems. Similarly, changes were made to the ordering of questions about veterinary opinions about animal health. In general, the variable most likely to be dependent, was positioned prior to any possibly corresponding independent variable. Various questions were also clarified and simplified. The final survey steps were those in Fig 1.

The final survey was made available from May–December 2020. It was widely advertised through social media to dog and cat interest groups. Paid Facebook advertising and several volunteers were utilised to increase survey exposure. Facebook advertising demographics were unlimited, other than to include terms relating to dogs and cats. In anticipation of lower levels of unconventional diets, and the need to achieve group numbers sufficient for statistical analysis, volunteers and the authors made some efforts to reach unconventional pet food interest groups, as well as conventional dog and cat interest groups. However, by careful wording choice, no bias for or against any particular diet choice was implied within advertising materials, or within the survey questions or explanatory text.

### Statistical analysis

We reported demographic results for survey respondents, and for their dogs. With respect to mean dog ages, we used T-tests to explore differences between dietary groups. When significant differences were found, effect size interpretations were provided using the Cohen’s d statistic, with small, medium or large effects interpreted when |d| was close to 0.2, 0.5 and 0.8, respectively [30]. With respect to sex/neuter status, where chi-squared results indicated the existence of significant differences, we provided effect size interpretations using the Cramer’s V statistic, with small, medium or large effects interpreted when V was close to 0.2, 0.5 and 0.8, respectively [31]. Where such significant differences existed, we then compared each main dietary group combination, calculating p-values. Where these indicated significant differences between dietary groups, we provided odds ratios, indicating relative differences in the likeliness of outcomes, between dietary groups.

After initial examination of dog diets, we limited further analysis to dogs maintained on three main diets: conventional meat, raw meat, and vegan pet food. We excluded smaller dietary groups to avoid potentially substantial differences in variances between dietary groups of small and larger sizes, which could adversely affect our statistical analysis.

We investigated the impacts of these three main diet types on dog health. Guardians provided information about seven general indicators of health, and about the prevalence of 22 specific health disorders, as described previously. Similarly to our analysis of sex/neuter status, for the main dietary groups identified, we investigated associations between diet type and the seven general health indicators using chi-squared tests, reporting test statistics and p-values. Where chi-squared results indicated the existence of significant differences, we similarly provided effect size interpretations using the Cramer’s V statistic, with small, medium or large effects interpreted when V was close to 0.2, 0.5 and 0.8, respectively [31]. Where such significant differences existed, we then compared each main dietary group combination, calculating p-values. Where these indicated significant differences between dietary groups, we provided odds ratios, indicating relative differences in the likeliness of outcomes, between dietary groups.

For most general health indicators, our data were categorical. However, assessments or opinions of health status were ordinal. These data were coded into levels 1 to 4 (indicating no health problems, up to seriously ill, respectively). We then used Kruskal-Wallis tests to explore differences between dietary groups. Dunn’s pairwise tests were carried out to explore differences within the three pairs of dietary comparisons, with the Bonferroni correction for multiple tests applied to p-values.

When investigating the significance of the association between diet and the number of disorders suffered by unwell dogs, we initially conducted an ANOVA test. Finding a significant difference, we calculated the effect size using eta squared. Following Cohen [32], we interpreted a small, medium or large effect depending on the proximity of eta squared to 0.01, 0.06 or 0.14 respectively. We also included a test for homogeneity of variance. Finding a lack of homogeneity, we then conducted a Games-Howell post hoc comparison to test the significance of apparent differences between dietary groups.

As well as exploring associations between diets and the seven general health indicators, we also explored associations with the 22 specific health disorders. Binomial response models provided odds ratios and p-values indicating differences between dietary groups. We used software packages R-studio and SPSS, v26. Significance was interpreted when p < 0.05.

### Ethical approval and data availability

Our research complied with the University of Winchester Ethics Policy [33] (approval reference RKEEC200304_Knight).

## Results

Of 4,060 respondents to our combined cat and dog survey, 4,057 confirmed they met the survey conditions (18 years or older, with answers relating to one dog or cat resident within their household, for at least one year). The following results are limited to the 2,639 dogs and their guardians who responded. Results concerning 1,418 cats and their guardians are the subject of a related, forthcoming study.

### Dog guardians

Of the 2,610 human respondents who provided their sex, 92% (2,412) identified as females, 7% (194) as males, and 0% (4) as other. Most age brackets from 18 to 70+ were well represented, other than the extreme ends where numbers were low. The majority of the 2,639 total respondents identified their geographical region as the UK (71%, 1,884) or Europe (15%, 398), with North America (6%, 150) and Australia/New Zealand/Oceania (4%, 117) being the next most prevalent continental regions. A minority (18%, 488/2,639) worked in the pet or veterinary industries. The most common diet reportedly followed by these 2,639 survey respondents was omnivorous (40%, 1,066), followed by vegan (22%, 586), reducetarian (omnivore reducing animal product consumption) (21%, 567), vegetarian (10%, 266) and pescatarian (consuming fish but no other meats) (5%, 134).

#### Importance of health to guardians

Of the 2,612 respondents who indicated their involvement in pet diet decision-making, 95% (2,489) were primary decision-makers, 4% (107) played some lesser role, and 1% (16) played no role. Those 99% (2,596) playing at least some role were asked which factors were important when choosing pet diets. Among 13 options including ‘other’, health and nutrition was considered the most important factor, being of importance to 94% (2,453) of 2,596 respondents to this question. These 2,596 individuals were asked which health and nutrition factors were important to them. Maintenance of pet health was considered the most important factor among five health and nutrition options including ‘other’. It was cited as important by 90% (2,211) of 2,449 respondents to this question.

The importance of health was similarly highlighted by the 1,370 respondents who used a conventional meat formulation as their dog’s normal diet, and the 830 who used a raw meat formulation. These combined 2,200 respondents were asked whether they would realistically choose alternative diets, if these offered their desired attributes. The alternatives offered for consideration were vegetarian and vegan diets, as well as those based on laboratory grown meat, insects, fungi and algae. Of 2,181 who answered this question, 44% (955) confirmed they would realistically choose such alternative diets. ‘Confidence about pet health’ was the second most important among 14 desired attributes (including ‘other’), that any alternative diet would need to provide. It was cited as essential by 83% (789) of these 955 respondents, after ‘Confidence about nutritional soundness’ (84%, 805).

### Dogs

#### Diets

**Fig 2:**
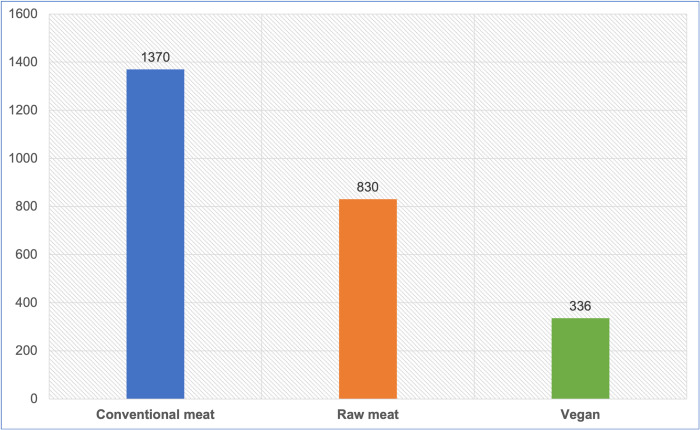
Three main diets fed to 2,536 dogs.

2,639 dog guardians responded, each describing a single dog. 2,612 indicated the main diet their dog was maintained on. 2,536 dogs were jointly maintained on the three main diets identified. These were conventional meat (1,370–54%), raw meat (830–33%) and vegan (336–13%) diets (Fig 2). Smaller dietary groups were excluded from further analysis. The largest excluded group was dogs reportedly fed vegetarian diets (n = 35). We also excluded 41 dogs reportedly maintained on fungi- (1) and insect-based diets (6), laboratory-grown meat (7), mixtures of other dietary types (17) and diets listed as ‘unsure’ (10). These groups were excluded due to low numbers, lack of clarity concerning the main ingredient type, or current unavailability of these sources as canine maintenance diets (as distinct from treats, snacks or supplements). Included within the set of 2,612 were 46 dog diets identified as ‘other’. These were examined and reclassified into conventional meat, mixture or unsure, depending on further details provided in textual answers.

As mentioned, we chose not to exclude dogs who received treats regularly, expecting most would fall within this group. This turned out to be true, with 76% (1,935) of these 2,536 dogs receiving treats/snacks/scraps at least once daily. Treats provided to these 2,536 dogs were most commonly vegetables or fruit (1,315), commercial treats (1,174), dental/oral bars or chewable sticks (1,129), human food prepared at home (901), and raw meat or bones (758). Some dogs received more than one kind of treat.

Thirty seven percent (926) of these 2,536 dogs were also regularly offered dietary supplements other than treats/snacks/scraps. These included products for joint health (558), fatty acids (e.g., omega-3 fatty acids) (364), probiotics or prebiotics (349), vitamins (235), minerals (198), digestive enzymes (130), amino acids (101) such as taurine, and other products. Some dogs received more than one kind of supplement.

#### Ages

**Fig 3:**
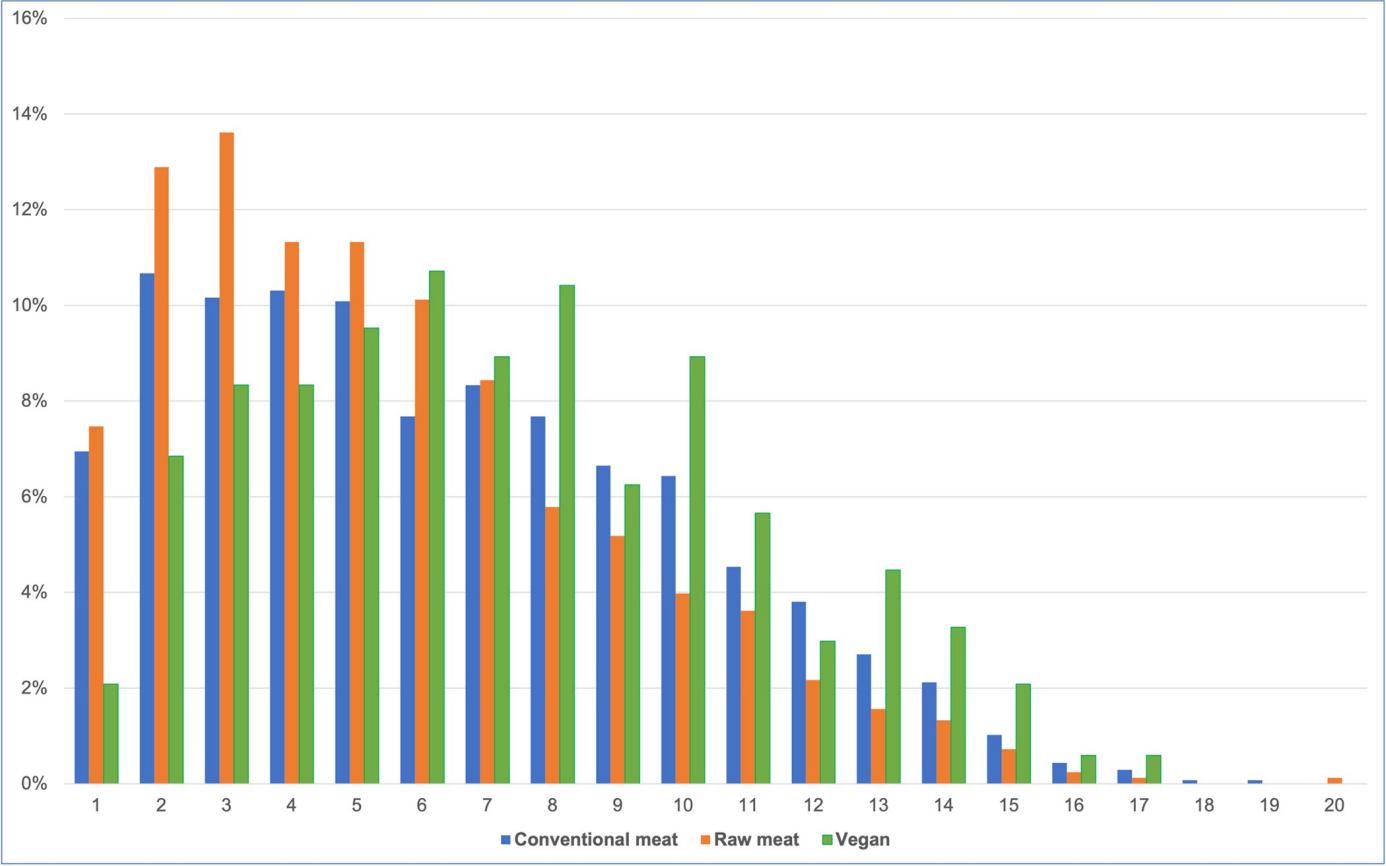
Ages of 2,534 dogs fed three main diets.

Considering the 2,536 dogs fed the three main diets, guardians were unsure of dogs’ ages in two cases. Ages of the remaining 2,534 dogs are indicated in Fig 3. The mean ages in years, were: overall– 6.18, raw meat– 5.52, conventional meat– 6.31, vegan– 7.30. Differences between all diet groups were significant, and of small to medium size (Table 1).

**Table 1:** Age differences between 2,534 dogs fed three main diets.

Age	Conventional—Raw meat	Conventional—Vegan	Raw meat—Vegan
T-value	-5.078	4.439	7.672
P-value	0.000	0.000	0.000
Cohen’s d	-0.218	0.268	0.513
Effect size	small	small	medium

#### Sex/Neuter status

**Fig 4:**
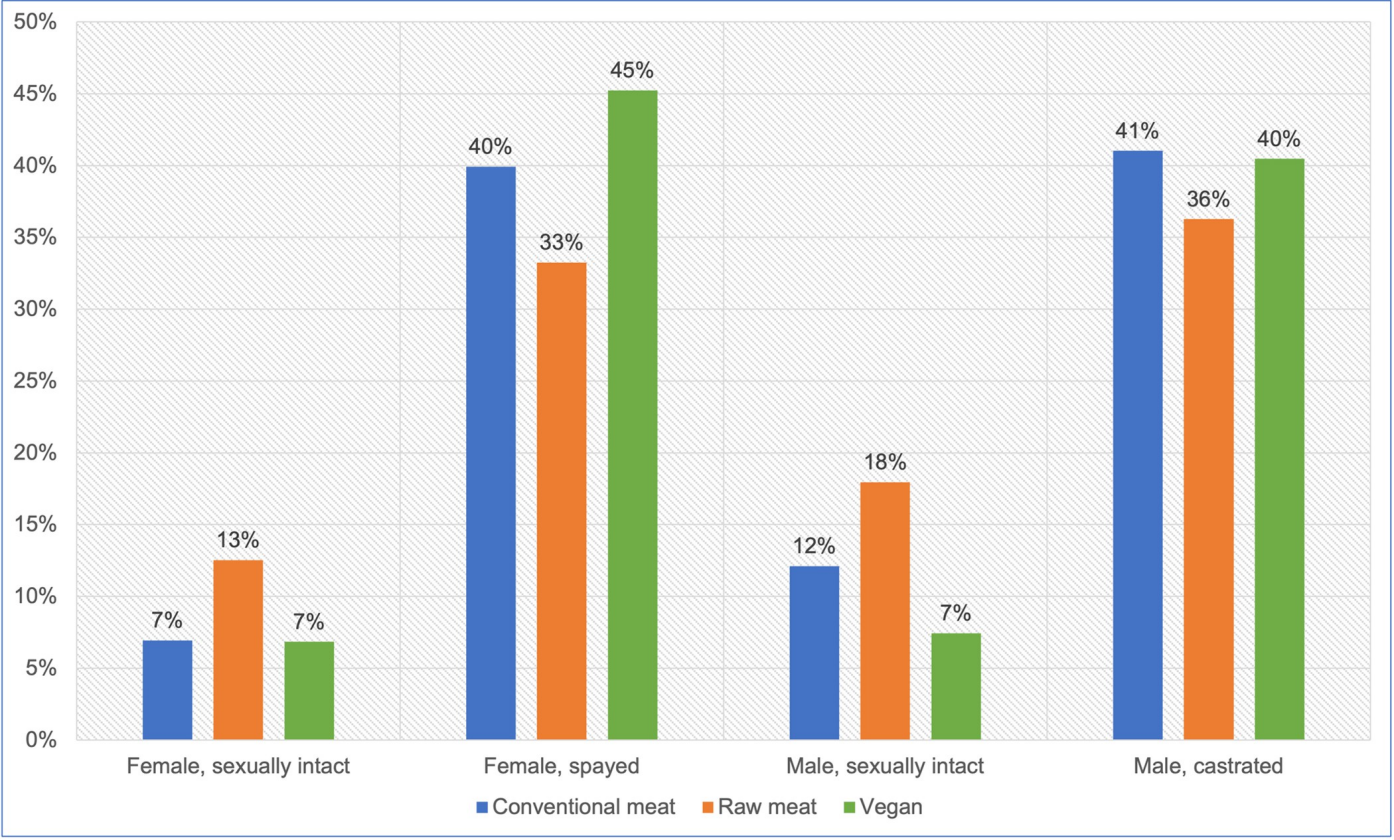
Sex/neuter status of 2,536 dogs fed three main diets.

The sex/neuter status of these 2,536 dogs is provided in Table 2 and Fig 4. Females comprised around 47%, and males around 53% of this sample. A chi-square test of independence showed a significant association between diet type and sex/neuter status, overall: χ2 (6) = 57.23, p < 0.05. The effect size was small (Cramer’s V = 0.106). Within this sample, dogs fed vegan diets were slightly more likely to be female, than either of the other two dietary groups. However, sex and diet type were not statistically significantly associated: χ2(2) = 3.9468, p = 0.139.

**Table 2:** Sex/neuter status of 2,536 dogs fed three main diets.

Sex/neuter status	Conventional meat	Raw meat	Vegan	Total
Female, sexually intact	95	104	23	222
Female, spayed	547	276	152	975
Male, sexually intact	166	149	25	340
Male, castrated	562	301	136	999
**Total**	**1370**	**830**	**336**	**2536**
**P-value**	** **	** **	** **	** **
Conventional meat	---	0.000	0.043	
Raw meat	0.000	---	0.000	
Vegan	0.043	0.000	---	
**Odds ratio**				
Conventional meat	---	0.537	1.412	
Raw meat	1.863	---	2.631	
Vegan	0.708	0.380	---	

**Note:** p-values and odds ratios reflect the likelihood of dogs being sexually intact.

Statistically significant differences were apparent however, with respect to desexing. The odds of being sexually intact were significantly different between all dietary groups (Table 2). Dogs fed vegan diets were less likely, and dogs fed raw meat were more likely, to be sexually intact, than conventionally fed dogs. Dogs fed raw meat were more than twice as likely to be sexually intact, than dogs fed vegan diets. Additionally, the odds of being sexually intact were significantly different between males and females, with males significantly more likely to be sexually intact (Table 3).

**Table 3:** Differences in odds of being sexually intact, between males and females, for 2,536 dogs fed three main diets.

	Male	Female
**P-value**		
Male	---	0.000
Female	0.000	---
**Odds ratio**		
Male	---	1.495
Female	0.669	---

### General health indicators

The results in this section consider the 2,536 dogs in the three main dietary groups.

#### Number of veterinary visits

**Fig 5:**
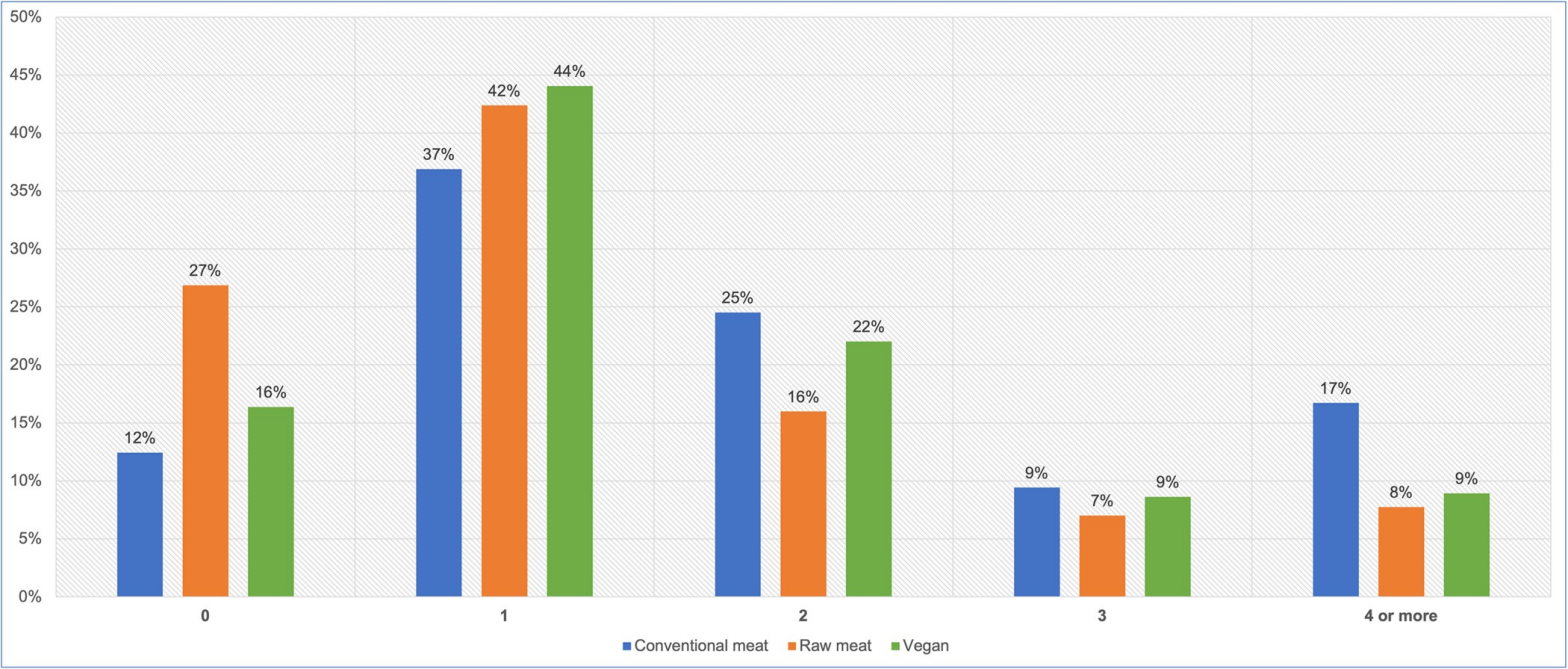
Veterinary visits of 2,520 dogs fed three main diets, in the last year.

After excluding 16 ‘unsure’ responses, 2,520 guardians reported frequency of veterinary visits within the last year (Fig 5, Table 4). Routine health checks are normally conducted annually, whereas multiple veterinary visits within a single year may sometimes indicate a health problem. We were interested in those dogs who saw veterinarians more than once in the previous year. A chi-square test of independence showed that there was a significant association between diet type and veterinary visits more or less than once in the last year: χ2 (2) = 84.75, p < 0.05. The effect size was small (Cramer’s V = 0.183).

**Table 4:** Veterinary visits of 2,520 dogs fed three main diets, in the last year.

Veterinary visits in the last year	Conventional meat	Raw meat	Vegan	Total
0	169	222	55	446
1	501	350	148	999
2	333	132	74	539
3	128	58	29	215
4 or more	227	64	30	321
**Total**	**1358**	**826**	**336**	**2520**

There were significant differences (p < 0.05) in the likelihood of seeing veterinarians more than once in the previous year, between all dietary groups (Table 5). Dogs fed vegan diets were less likely, and dogs fed raw meat less than half as likely, to meet this criterion, than conventionally fed dogs. Dogs fed vegan diets were more likely to meet this criterion, than dogs fed raw meat.

**Table 5:** Likelihood of seeing veterinarians more than once in the previous year, for 2,520 dogs fed three main diets.

	Conventional meat	Raw meat	Vegan
**P-value**			
Conventional meat	---	0.000	0.000
Raw meat	0.000	---	0.004
Vegan	0.000	0.004	---
**Odds ratio**			
Conventional meat	---	2.312	1.567
Raw meat	0.432	---	0.678
Vegan	0.638	1.475	---

#### Medication use

**Fig 6:**
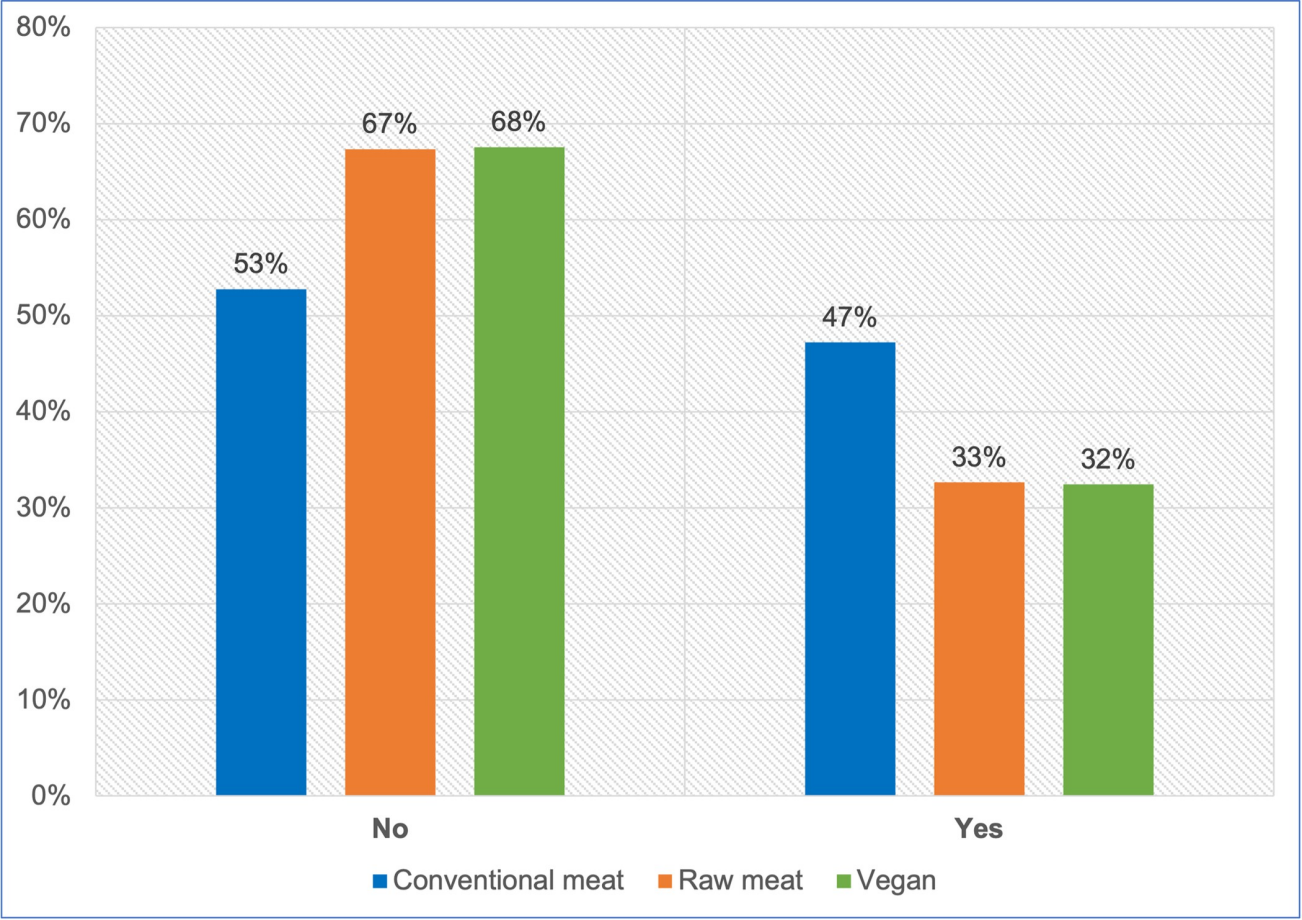
Medication use in 2,536 dogs fed three main diets.

All 2,536 guardians provided information about medication use in the previous year (Fig 6, Table 6). A chi-square test of independence showed a significant association between diet type and medication use: χ2 (2) = 56.002, p < 0.05. The effect size was small (Cramer’s V = 0.149). There were significant differences (p < 0.05) in the likelihood of medication usage in the previous year, between dogs fed vegan and conventional diets, and between dogs fed raw meat and conventional diets, but not between dogs fed vegan and raw meat diets. Dogs fed vegan and raw meat diets each had a lower risk of meeting this criterion, compared to conventionally fed dogs.

**Table 6:** Medication use in 2,536 dogs fed three main diets.

Medication use	Conventional meat	Raw meat	Vegan	Total
No	723	559	227	1509
Yes	647	271	109	1027
**Total**	**1370**	**830**	**336**	**2536**
**P-value**	** **	** **	** **	
Conventional meat	---	0.000	0.000	
Raw meat	0.000	---	0.945	
Vegan	0.000	0.945	---	
**Odds ratio**	** **	** **	** **	
Conventional meat	---	1.846	1.863	
Raw meat	0.542	---	1.010	
Vegan	0.537	0.990	---	

**Note:** p-values and odds ratios reflect the likelihood of medication being used.

#### Progression onto a therapeutic diet

**Fig 7:**
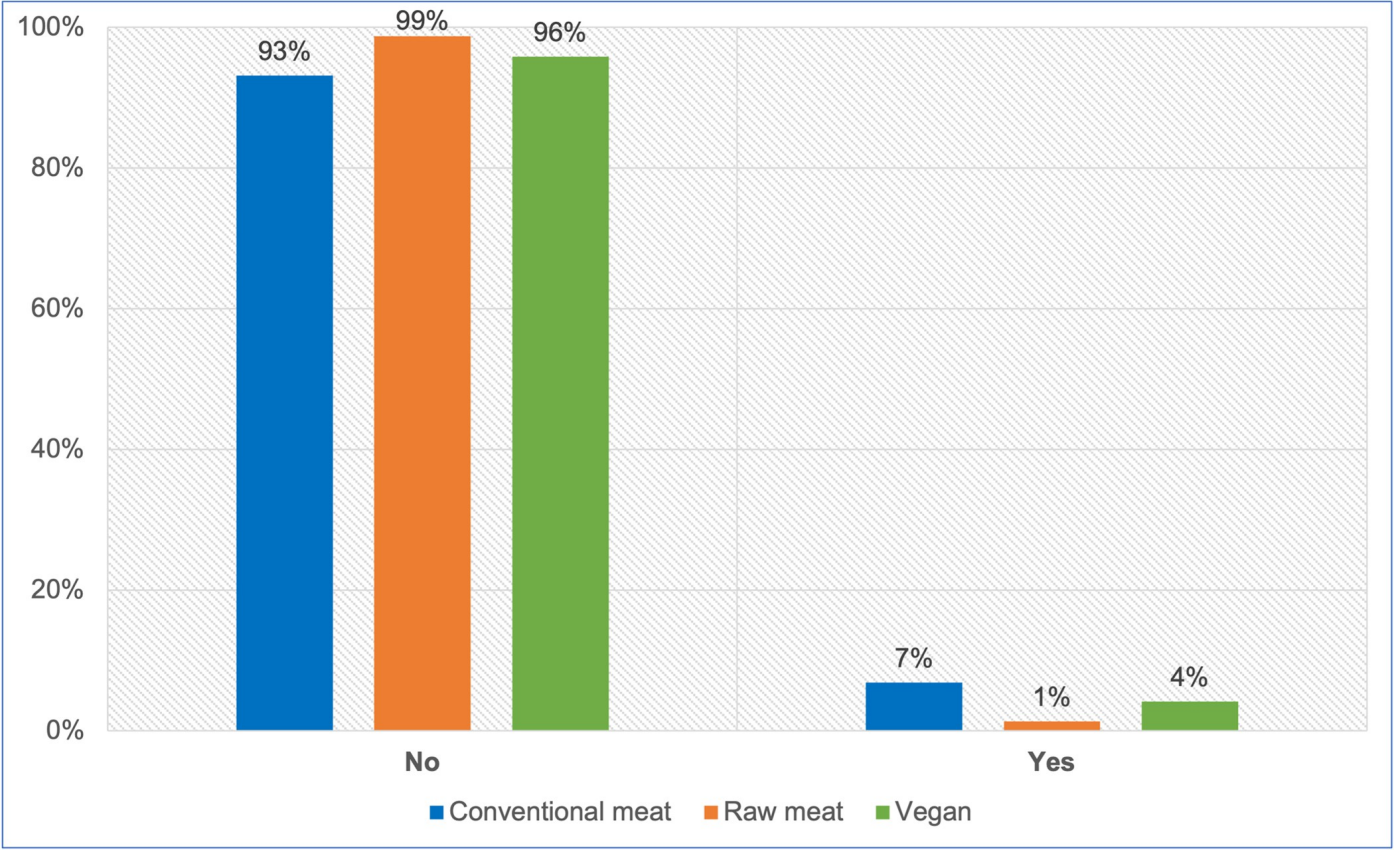
Subsequent progression onto a therapeutic diet in 2,536 dogs maintained on an initial diet as specified.

All 2,536 guardians provided information about whether or not their dog progressed onto a therapeutic diet, after initial maintenance on one of the three main diets (Fig 7, Table 7). A chi-square test of independence showed a significant association between initial diet type and subsequent progression onto a therapeutic diet: χ2 (2) = 35.659, p < 0.05. The effect size was small (Cramer’s V = 0.119). There were significant differences (p < 0.05) in likelihood of subsequent progression onto a therapeutic diet, between dogs initially fed raw meat and conventional diets, and between dogs initially fed vegan diets and raw meat, but not between dogs initially fed vegan and conventional diets. Dogs initially fed raw meat were less than one fifth as likely to meet this criterion, as dogs initially fed conventional diets, and dogs initially fed vegan diets had more than three times the risk of this outcome, compared to those initially fed raw meat.

**Table 7:** Subsequent progression onto a therapeutic diet in 2,536 dogs maintained on an initial diet as specified.

Progressed to therapeutic diet	Conventional meat	Raw meat	Vegan	Total
No	1276	819	322	2417
Yes	94	11	14	119
**Total**	**1370**	**830**	**336**	**2536**
**P-value**	** **	** **	** **	
Conventional meat	---	0.000	0.072	
Raw meat	0.000	---	0.004	
Vegan	0.072	0.004	---	
**Odds ratio**	** **	** **	** **	
Conventional meat	---	5.485	1.694	
Raw meat	0.182	---	0.309	
Vegan	0.590	3.237	---	

**Note:** p-values and odds ratios reflect the likelihood of subsequent progression onto a therapeutic diet.

#### Reported veterinary assessments of health status

**Fig 8:**
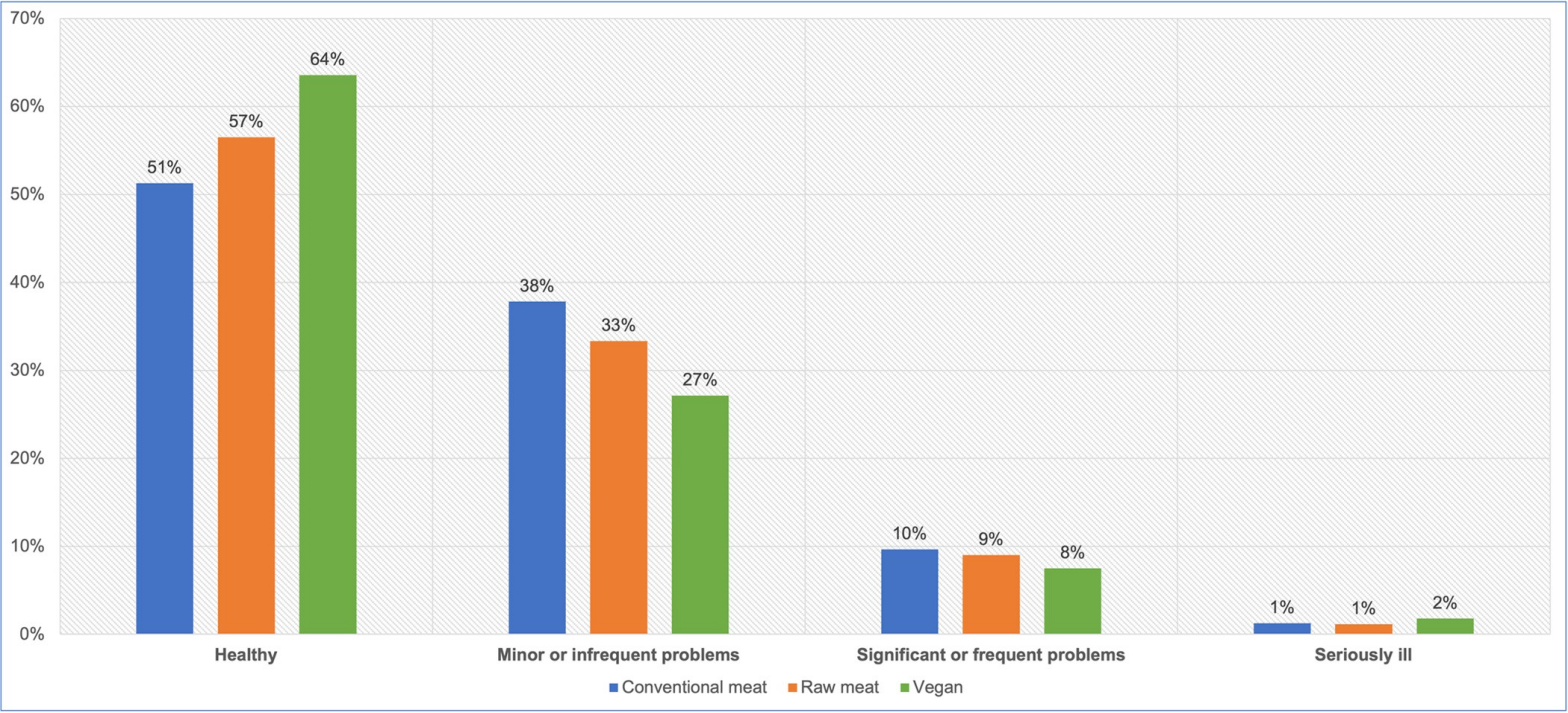
Guardian-reported veterinary assessments of the health status of 2,062 dogs fed three main diets.

2,074 dogs saw a veterinarian at least once in the previous year (Table 4). After excluding 12 ‘unsure’ respondents, the 2,062 remaining guardians were reportedly sure of the assessments of their veterinarians regarding the health status of their dogs (Fig 8, Table 8). A chi-square test of independence showed a significant association between diet type and reported veterinary assessment: χ2 (6) = 16.770, p = 0.0101. The effect size was small (Cramer’s V = 0.064).

**Table 8:** Guardian-reported veterinary assessments of the health status of 2,062 dogs fed three main diets.

Reported veterinary assessments	Conventional meat	Raw meat	Vegan	Total
No problems or routine preventative healthcare	606	339	178	1123
Minor or infrequent problems	447	200	76	723
Significant or frequent problems	114	54	21	189
Seriously ill	15	7	5	27
**Total**	**1182**	**600**	**280**	**2062**

After coding into 1 to 4 (indicating no health problems (1), up to seriously ill (4), respectively), significant differences existed between dogs fed vegan and conventional meat diets, but not between other dietary groups. A Kruskal-Wallis test provided very strong evidence of a difference (p = 0.002) between the means ranks of at least one pairing (Table 9). Dunn’s pairwise tests were carried out for the three pairs of groups. There was very strong evidence (p = 0.002, adjusted using the Bonferroni correction) of a difference between dogs fed a vegan and a conventional meat diet. There was no evidence of differences between dogs fed vegan and raw meat diets, or for dogs fed raw or conventional meat diets (Table 10).

**Table 9:** Differences in guardian-reported veterinary assessments of the health status of 2,062 dogs fed three main diets.

	Conventional meat	Raw meat	Vegan
Mean Rank	1062.92	1011.13	942.51
Kruska-Wallis H	12.901		
df	2		
P-value	0.002		

**Table 10:** Pairwise comparison of guardian-reported veterinary assessments of the health status of 2,062 dogs fed three main diets.

Diet 1 –Diet 2	Test Statistic	Std Error	Std Test Statistic	P-value	Adjusted P-value^1^
Conventional—Raw meat	51.799	26.604	1.947	0.052	0.155
Conventional—Vegan	120.417	35.274	3.414	0.001	0.002
Raw meat—Vegan	68.618	38.411	1.786	0.074	0.222

^1^ Significance values have been adjusted by the Bonferroni correction for multiple tests.

When comparing each main diet group combination and calculating odds ratios, there were significant differences (p < 0.05) in the likelihood that reported veterinary assessments of health status would indicate poorer health, between dogs fed vegan and conventional diets, but not between the other dietary groups (Table 11). Dogs fed vegan diets were less likely to meet this criterion, than conventionally fed dogs.

**Table 11:** Likelihood of guardian-reported veterinary assessments indicating poorer health, in 2,062 dogs fed three main diets.

	Conventional meat	Raw meat	Vegan
**P-value**			
Conventional meat	---	0.053	0.001
Raw meat	0.053	---	0.064
Vegan	0.001	0.064	---
**Odds ratio**			
Conventional meat	---	1.208	1.585
Raw meat	0.828	---	1.312
Vegan	0.631	0.762	---

#### Guardian opinions of health status

**Fig 9:**
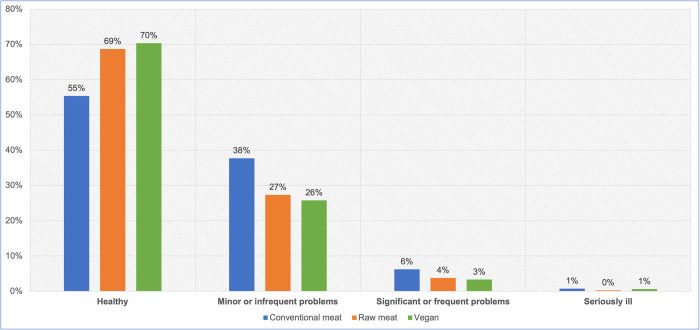
Guardian opinions of the health status of 2,530 dogs fed three main diets.

After excluding six ‘unsure’ responses, 2,530 guardians reported their own opinions of the health status of their dogs (Fig 9, Table 12). A chi-square test of independence showed a significant association between diet type and guardian opinion: χ2 (6) = 52.875, p < 0.05. The effect size was small (Cramer’s V = 0.102).

**Table 12:** Guardian opinions of the health status of 2,530 dogs fed three main diets.

Guardian opinions	Conventional meat	Raw meat	Vegan	Total
Healthy	758	568	235	1561
Generally healthy with minor or infrequent problems	516	226	86	828
Significant or frequent problems	85	31	11	127
Seriously ill	10	2	2	14
**Total**	**1369**	**827**	**334**	**2530**

After coding into 1 to 4 (indicating no health problems (1), up to seriously ill (4), respectively), statistical analysis indicated significant differences between dogs fed vegan and conventional diets. A Kruskal-Wallis test provided very strong evidence of a difference (p < 0.0001) between the means ranks of at least one pairing (Table 13). Dunn’s pairwise tests were carried out for the three pairs of groups. There was very strong evidence (p < 0.0001, adjusted using the Bonferroni correction) of differences between dogs fed vegan and conventional meat diets, and between dogs fed conventional meat and raw meat diets. There was no evidence of a difference between dogs fed vegan and raw meat diets (Table 14).

**Table 13:** Differences in guardian opinions of the health status of 2,530 dogs fed three main diets.

	Conventional meat	Raw meat	Vegan
Mean Rank	1347.77	1174.32	1154.07
Kruska-Wallis H	52.088		
df	2		
P-value	< 0.0001		

**Table 14:** Pairwise comparison of guardian opinions of the health status of 2,530 dogs fed three main diets.

Diet 1 –Diet 2	Test Statistic	Std Error	Std Test Statistic	P-value	Adjusted P-value^1^
Conventional—Raw meat	173.451	27.487	6.310	< 0.0001	< 0.0001
Conventional—Vegan	193.703	38.088	5.086	< 0.0001	< 0.0001
Raw meat—Vegan	20.252	40.462	0.501	0.617	1.000

^1^ Significance values have been adjusted by the Bonferroni correction for multiple tests.

When comparing each main dietary group combination and calculating odds ratios, there were significant differences (p < 0.05) in the likelihood guardians would assess their dogs as having worse health, between dogs fed vegan and conventional diets, and between dogs fed raw meat and conventional diets, but not between dogs fed vegan and raw meat diets (Table 15). Dogs fed vegan and raw meat diets were both less likely to meet this criterion, compared to conventionally fed dogs.

**Table 15:** Likelihood of guardian opinions indicating poorer health, in 2,530 dogs fed three main diets.

	Conventional meat	Raw meat	Vegan
**P-value**			
Conventional meat	---	0.000	0.000
Raw meat	0.000	---	0.590
Vegan	0.000	0.590	---
**Odds ratio**			
Conventional meat	---	1.770	1.909
Raw meat	0.565	---	1.079
Vegan	0.524	0.927	---

### Specific disorders

2,074 dogs saw veterinarians at least once in the previous year (Table 4). After excluding 12 cases in which guardians were unsure what veterinary opinions would be, guardians were reportedly sure of the opinions of 2,062 veterinarians (Table 8). 1,123 of these dogs were considered entirely healthy. The remaining 939 dogs were considered to suffer from one or more disorders. In eight cases (conventional meat– 3, raw meat– 4, vegan– 1), details were not provided or veterinarians reportedly considered dogs to be ‘healthy’, ‘old’, or variations of these–i.e. not truly unwell. These dogs were excluded. The remaining 931 dogs were analysed. In 161 of these cases, details of ‘other’ disorders were reported. These were examined, and then reclassified into the 18 existing, or four new disorder types. In total, respondents reported that these 931 dogs were considered by their veterinarians to be suffering from 1,477 cases of 22 specific disorders (S1 Table).

For five disorders, guardians had the option to provide additional information. With respect to skin/coat problems, further information was provided about 140 of these 147 cases. The most common causes, in order, were atopic/allergic dermatitis (inflamed skin due to allergies), moist dermatitis, and pruritis (itchiness) of unspecified origin. With respect to mobility problems, further information was provided about 123 of these 135 cases. The most common causes, in order, were osteoarthritis/arthritis, and a variety of ’other’ causes. With respect to dental/oral problems, further information was provided about 109 of these 110 cases. The most common causes, in order, were dental calculus/plaque/tartar, gingivitis, and a variety of ‘other’ causes, particularly damaged, broken or worn teeth. With respect to body weight problems, all 80 respondents described whether dogs were over- or underweight. Eighty five percent (68) of reported cases were overweight, and 15% (12) were underweight. With respect to eye problems, further information was provided about 57 of these 58 cases. The most common causes included eye ulcers and related conditions such as dry eye and entropion, conjunctivitis, infections, blindness/vision loss and cataracts.

#### Percentage of unwell dogs and average number of disorders per unwell dog

In addition to these 931 dogs with 1,477 cases of 22 specific disorders, respondents reported that the remaining 1,123 dogs were considered by their veterinarians to be healthy. Overall, 45% were suffering from at least one disorder, and the average number of disorders per unwell dog was 1.59 (Table 16).

**Table 16:** 1,477 occurrences of 22 specific disorders, in 2,054 dogs fed three main diets.

Health status	Conventional meat	Raw meat	Vegan	Total
Unwell	573	257	101	931
Healthy	606	339	178	1123
**Total dogs**	**1179**	**596**	**279**	**2054**
**% unwell**	**49%**	**43%**	**36%**	**45%**
Cases of disorders	947	377	153	1477
**Average cases/unwell dog**	**1.65**	**1.47**	**1.51**	**1.59**
**P-value**	** **	** **	** **	** **
Conventional meat	---	0.029	0.000	** **
Raw meat	0.029	---	0.052	** **
Vegan	0.000	0.052	---	** **
**Odds ratio**	** **	** **	** **	** **
Conventional meat	---	1.247	1.666	** **
Raw meat	0.802	---	1.336	** **
Vegan	0.600	0.748	---	** **

**Note:** p-values and odds ratios reflect the likelihood of dogs being assessed as unwell.

#### Percentage of unwell dogs

A chi-square test of independence showed that there was a statistically significant association between diet type and the number of unwell dogs: χ2 (2) = 15.65, p < 0. 0001. The effect size was small (Cramer’s V = 0.087). There were significant differences (p < 0.05) in the likelihood of being considered unwell between dogs fed vegan and conventional meat diets, and between dogs fed raw and conventional meat diets, but not between dogs fed vegan and raw meat diets. Dogs fed both vegan and raw meat diets had less risk of this outcome compared to conventionally fed dogs (Table 16).

#### Number of disorders per unwell dog

The number of disorders per unwell dog ranged from one to eight (Table 17). An ANOVA test revealed a significant difference between the number of disorders suffered by unwell dogs, depending on diet type (F = 3.953 (2, 928), p = 0.02). The effect size was small (eta squared = 0.008). A test of homogeneity of variances indicated that variances were not homogenous. Accordingly, a Games-Howell post hoc comparison test was used. This revealed that unwell dogs fed a raw meat diet suffered less disorders than unwell dogs fed a conventional meat diet. This difference was statistically significant (p = 0.022). Unwell dogs fed a vegan diet did not significantly differ in the number of disorders suffered, compared to unwell dogs fed conventional or raw meat diets.

**Table 17:** Number of disorders per unwell dog, among 931 unwell dogs fed three main diets.

Number disorders per dog	1	2	3	4	5	6	7	8
Conventional meat	330	160	50	21	7	2	2	1
Raw meat	181	54	12	5	1	1	3	0
Vegan	64	25	10	1	1	0	0	0

#### Prevalence of 22 specific disorders

**Fig 10:**
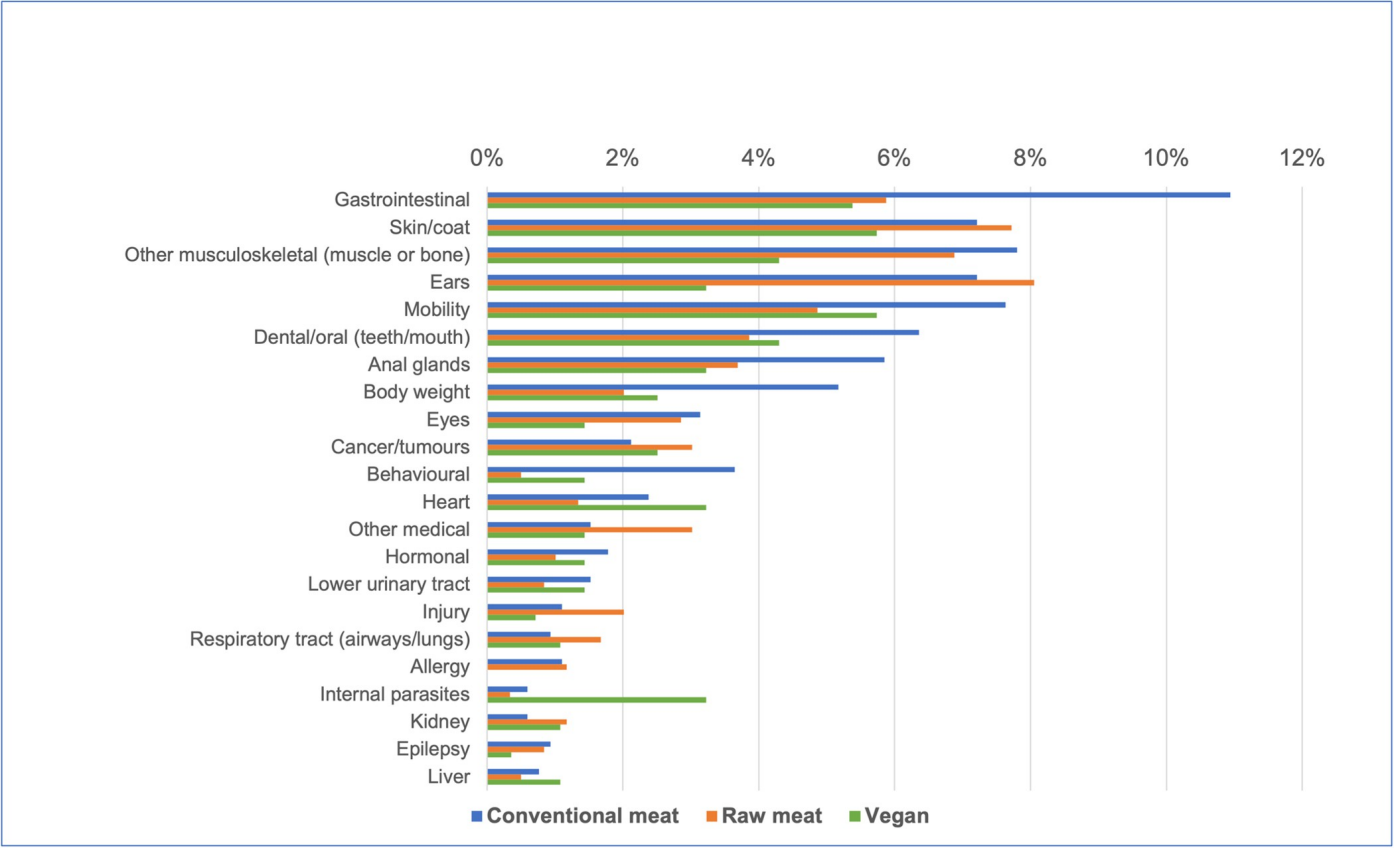
Prevalence of 22 specific disorders or affected bodily systems in 2,054 dogs fed three main diets, based on reported assessments of veterinarians. Vertical axis order reflects overall prevalence of disorders (combining all diets).

The prevalence of these 22 specific disorders in these 2,054 dogs is indicated in S2 Table and Fig 10.

#### Differences between dietary groups

Based on probability of occurrence, the 10 most common disorders found within each dietary group are listed in Table 18. Some significant differences in the prevalence of certain disorders between dietary groups were detected. There are indicated in S3 Table and summarised in Table 19.

**Table 18:** The 10 most common disorders or affected bodily systems among 2,054 dogs fed three main diets, and overall, based on reported assessments of veterinarians.

Rank	Conventional meat	Raw meat	Vegan	Overall
1	Gastrointestinal (e.g., diarrhoea, vomiting) (11%)	Ears (8%)	Skin/coat (6%)	Gastrointestinal (e.g., diarrhoea, vomiting) (9%)
2	Other musculoskeletal (muscle or bone) disease (8%)	Skin/coat (8%)	Mobility (6%)	Skin/coat (7%)
3	Mobility (8%)	Other musculoskeletal (muscle or bone) disease (7%)	Gastrointestinal (e.g., diarrhoea, vomiting) (5%)	Other musculoskeletal (muscle or bone) disease (7%)
4	Skin/coat (7%)	Gastrointestinal (e.g., diarrhoea, vomiting) (6%)	Other musculoskeletal (muscle or bone) disease (4%)	Ears (7%)
5	Ears (7%)	Mobility (5%)	Dental/oral (teeth/mouth) (4%)	Mobility (7%)
6	Dental/oral (teeth/mouth) (6%)	Dental/oral (teeth/mouth) (4%)	Ears (3%)	Dental/oral (teeth/mouth) (5%)
7	Anal glands (6%)	Anal glands (4%)	Anal glands (3%)	Anal glands (5%)
8	Body weight (5%)	Cancer/tumours (3%)	Heart (3%)	Body weight (4%)
9	Behavioural (4%)	Other medical (3%)	Internal parasites (3%)	Eyes (3%)
10	Eyes (3%)	Eyes (3%)	Cancer/tumours (3%)	Cancer/tumours (2%)

**Note:** Percentages provide the prevalence of each disorder within each dietary group, and overall (all diets combined).

**Table 19:** Disorders or bodily system effects with significantly different prevalences between dietary groups, among 2,054 dogs, based on reported assessments of veterinarians.

Conventional–Raw meat	Conventional—Vegan	Raw meat—Vegan
Raw less likely (5): Gastrointestinal, Mobility, Dental/oral, Body weight, Behavioural	Vegan less likely (3): Gastrointestinal, Other musculoskeletal, Ears	Vegan less likely (1): Ears
Raw more likely (1): Other medical	Vegan more likely (1): Internal parasites	Vegan more likely (1): Internal parasites

## Discussion

### Importance of health to guardians

Our results affirmed the importance of pet health to guardians. Among 2,596 respondents, health and nutrition was the factor considered most important in purchasing decisions. These results concur with other studies. ‘Health & Nutrition’ was the most important among 24 pet food characteristics ranked by 2,181 pet guardians, in a US-based study from 2015 to 2016 by Schleicher and colleagues [34].

It was noteworthy that 44% of our respondents feeding conventional or raw meat-based diets, stated they would realistically consider alternatives. These results were similar to those of Dodd and colleagues [35], who surveyed 3,673 primarily Canadian and US pet guardians. They found that 35% (1,083/3,130) of responding guardians who did not already feed a plant-based diet to their dog or cat, indicated interest in doing so. Our respondents reported that the most important attributes such an alternative diet would need to provide, were ‘confidence about nutritional soundness’, closely followed by ‘confidence about pet health’ (cited as necessary by 84% and 83% of these respondents, respectively). Dodd and colleagues found that the most important attributes such an alternative diet would need to provide, were ‘further evidence of nutritional sufficiency’ (45% - 269/599), followed by veterinary approval (20% - 122/599), and greater availability (20% - 117/599).

### Canine demographics

Health outcomes for animals fed different diets may be affected by age, sex and desexing (neutering) status [26, 36], and so it was important for us to understand whether these varied between different diets, and how they compared to normal dog populations, in our sampled dogs.

All three of the dietary groups we studied (conventional meat, raw meat, vegan) appeared to have had an age distribution broadly representative of normal dogs [26, 37], with the exception of the first five years of life, in which there was a higher percentage of dogs fed raw meat diets, and a lower percentage fed vegan diets (Fig 3). This resulted in significant differences between the average ages of these dietary groups. Within our studied sample, on average, the youngest dogs were those fed raw meat, and the oldest dogs were those fed vegan diets, with statistically significant differences between all dietary groups. Given that younger dogs generally have fewer health problems, this may have positively influenced the general health outcomes of dogs fed raw meat diets. In contrast, dogs fed vegan diets could be expected to have relatively worse health outcomes. Due to their shorter lifespans, a single canine year of life equates to many years of human life [38], from a veterinary healthcare perspective.

Females comprised just under half and males just over half of our 2,536 dogs (Table 2). This was consistent with a 2016 study of 22,333 UK dogs [26], which found just over half to be male. Variations in sex distributions between dietary groups within our sample were not statistically significant. Statistically significant differences did exist with respect to desexing. Around three quarters of all dogs in our sample were desexed (Table 2). This differed from the findings of O’Neill and colleagues [26], who found 45% of all dogs to be desexed. Within our sample, male dogs were more likely than females to be sexually intact (Table 3). This was consistent with the findings of O’Neill and colleagues, who found desexing to be slightly less common for males. We also found that desexing was most common for dogs fed vegan diets, and least common for dogs fed raw meat diets. Significant differences existed between all dietary groups in this respect (Table 2). Lowered desexing rates in guardians feeding raw meat diets may relate to the reduced likelihood of such guardians visiting veterinarians (Fig 5). Such guardians may be less likely to receive, or to comply with, veterinary advice, and routine preventative healthcare advice commonly includes desexing recommendations.

### General health indicators

#### Number of veterinary visits

At least in the UK, routine health checks and administration of preventative healthcare, such as vaccinations, are normally conducted annually [39–41]. Seventy-one per cent of our respondents were based in the UK. Visit numbers may increase somewhat for puppies or geriatric animals, but these comprised a low proportion of studied animals (Fig 3). Hence, zero or one veterinary visits in the previous year would normally be consistent with good health, for our sample. In contrast, two, three or more visits could indicate a health concern. Dogs fed conventional meat diets appeared more likely to fall within the latter group, than those fed raw meat or vegan diets (Fig 5). Effects were most notable for dogs fed raw meat diets, who were less than half as likely as conventionally fed dogs, to experience two or more veterinary visits (Table 5).

The apparent difference of raw meat diets in this respect, appears to have been heavily influenced by a large increase in the proportion of dogs who did not see a veterinarian at all in the last year, compared to the other two dietary groups. For dogs fed raw meat, these comprised 27%, compared to those fed conventional (12%) and vegan (16%) diets respectively (Fig 5). The unusually high proportion of dogs fed raw meat diets, who did not see a veterinarian at all within the last year, may indeed indicate a lack of healthcare problems. However, there is reason to believe that guardians of dogs fed raw meat are less likely to visit veterinarians, for reasons not directly related to the health of their animals. The overwhelming majority of veterinarians are critical of guardian choices to feed raw meat, due to well-documented concerns about nutritional soundness and pathogen contamination [42–51]. It is known that those who feed a raw meat diet are less likely to seek advice from their veterinarian, and more inclined to gather information from other sources, such as online resources [52]–which vary greatly in their reliability. The perceived opposition of most veterinarians to the feeding philosophy and choices of guardians feeding raw meat diets, may make these people less trusting of veterinary advice, and less likely to visit veterinarians, in general. This is likely to have altered this apparent general health indicator, for reasons unrelated to the health of these dogs.

#### Medication use

Medication use was similarly considered to indicate a probable health concern. This was significantly more prevalent among dogs fed conventional meat diets, compared with those fed raw meat or vegan diets (Fig 6, Table 6). Veterinary clinics are major sources of companion animal medications, and nearly the only source of prescription medications. It is a requirement in most jurisdictions, that animals receiving prescription medications be examined at least once by a veterinarian, within the preceding year. Accordingly, the markedly decreased proportion of veterinary visits by dogs fed raw meat, compared to other dietary groups (Fig 5), may have lowered the proportion of such dogs receiving medications in the previous year.

#### Progression onto a therapeutic diet

Guardians were asked whether their dog progressed onto a therapeutic diet, after being initially maintained primarily on a conventional meat, raw meat or vegan diet for at least one year. Such progression was also considered indicative of a possible health concern. This was reported by 5% (119/2536) of respondents, and was significantly more likely in dogs initially fed conventional and vegan diets, than in those initially fed raw meat (Table 7). As with medications, veterinary clinics are the major sources of therapeutic diets. Similarly to medication use, the markedly decreased proportion of veterinary visits by dogs fed raw meat, compared to other dietary groups (Fig 5), may have lowered the proportion of such dogs who received therapeutic diets. Additionally, to the authors’ knowledge, by late 2021 few therapeutic diets were marketed as ‘vegan’ and none as ‘raw meat’. Hence, even where dogs fed these diets were seen by veterinarians and recommended a therapeutic diet, it is possible guardians feeding these diets might not have complied with the recommendation.

#### Reported veterinary assessments of health status

When considering the veterinary healthcare assessments of their dogs, by their veterinarians, guardians of dogs fed conventional diets were significantly more likely to report that veterinarians considered dogs to be suffering from healthcare problems, than guardians of dogs fed vegan diets. Differences between other dietary groups were not statistically significant (Table 11).

#### Guardian opinions of health status

A similar pattern was revealed when guardians were asked for their own assessments of their dogs’ health status–albeit with a shift of roughly 5% in all groups, toward considering dogs to be healthier than veterinarians were expected to rate them (Fig 9). Guardians were significantly more likely to assess their dogs as having worse health, when fed conventional diets, compared to dogs fed vegan or raw meat diets. Differences between the latter two groups were not statistically significant (Table 15).

#### Percentage of unwell dogs

After limiting to dogs who had seen a veterinarian at least once in the previous year, and excluding dogs for whom guardians were unsure of their veterinarians’ assessments, and eight instances in which details were not provided or veterinarians reportedly did not consider dogs to be truly unwell, 2,054 dogs remained (Table 16). Forty five percent of these dogs were considered to suffer from at least one health disorder. This is lower than the 66% of 22,333 UK dogs reported by O’Neill and colleagues [26] to suffer from at least one disorder during 2016. This may be attributable to our active efforts to recruit participants feeding unconventional diets. Forty six percent of our sampled dogs were fed raw meat or vegan diets, and our combined results indicate that these dogs appeared to suffer from disorders less commonly than dogs fed conventional meat diets. The likelihood of being considered unwell, was significantly greater for dogs fed conventional diets compared to those fed raw meat or vegan diets, but there was no significant difference between dogs fed vegan and raw meat diets (Table 16).

#### Number of disorders per unwell dog

The number of health disorders per unwell dog varied from one to eight (Table 17), with the average number of disorders per unwell dog being 1.59 (Table 16). Unwell dog fed a raw meat diet suffered from fewer disorders than unwell dog fed a conventional meat diet, but differences between the other dietary groups were not statistically significant.

#### General health indicators overall

We compared the dogs in each diet group with those of the other two diet groups (Table 20). Those dogs fed conventional diets appeared to fare worse than those fed either of the other two diets.

**Table 20:** Performance of each dietary group compared to the other two diets, with respect to seven general indicators of health.

	**Conventional meat**	**Raw meat**	**Vegan**
Superior	0	8	5
Equivalent	3	6	7
Inferior	11	0	2

**Note**: Comparing each diet group with the other two diets, for seven general indicators of health, results in 14 comparisons; hence each column totals 14.

On the face of it, dogs fed raw meat appeared to fare slightly better than those fed vegan diets. However, there was a statistically significant, medium-sized difference between the average ages of dogs in these two groups. This is likely to have improved the general health indicators of dogs fed raw diets, and to have lowered the prevalence of certain specific disorders [26]. In our study dogs fed raw meat appeared less likely to suffer from certain specific disorders (Table 19)–one, when compared to dogs fed vegan diets, and five, when compared to dogs fed conventional diets. However, for at least three of these six (dental/oral, body weight and mobility disorders), the younger ages of dogs fed raw meat, is likely to have decreased the prevalence of these disorders [36].

Additionally, there appear to be reasons unrelated to health, which significantly decrease the likelihood guardians of dogs fed raw meat, will trust the opinions of their veterinarians, and seek veterinary visits. The proportion of dogs who never saw veterinarians in the last year was markedly higher for those fed raw meat, compared with those fed vegan or conventional diets (Fig 5). Decreased veterinary visits also affects the likelihood dogs will receive medication or progress to therapeutic diets. Jointly, these three health indicators comprise nearly half of the seven general health indicators studied.

In light of these potentially confounding factors, and that the effect size was statically small, for every general health indicator examined, we cannot conclude that dogs fed raw meat diets would be likely to have health outcomes superior to those fed vegan diets, if ages were equalised, and non-health related barriers to visiting veterinarians, were accounted for.

#### Consistency with prior related studies

When considering previous research in this field, by 2021 only Semp [20] had similarly published guardian-reported health outcomes in dogs. Some of those guardians reported a range of specific health benefits associated with vegan diets, as noted in the following (‘Specific disorders’). Semp and other investigators have also reported veterinary clinical examination and laboratory test results exploring the health of dogs maintained on vegan diets. Semp reported that clinical examinations and blood tests of 20 vegan dogs did not reveal any abnormalities associated with diet. Not even the 10% (2/20) of dogs fed a homemade supplemented diet showed any significant deviations.

Yamada and colleagues [18] conducted research on eight dogs, divided into two groups maintained on animal or vegetable protein-based diets. It was not clear whether the latter was a vegan diet. The VP-based dogs developed marked anaemia following exercise. However, the experimental regime was particularly severe: six weeks of rest followed by four hours daily of enforced running at 12 km/h, for two weeks. This deviates markedly from the normal experience of domesticated dogs, and so is of limited relevance to them. The sample size was also too small for reliable extrapolation of results to wider dog populations.

Brown and colleague’s 2009 study of sprint racing Siberian Huskies [19], did not record anaemia or other detectable health problems, in dogs fed either meat or VP-based diets (each n = 6) over 16 weeks, including 10 weeks of competitive racing. Both timeframe and sample size were larger than those used by Yamada and colleagues [18], although this sample size remained limited.

#### Specific disorders (22)

The ten most common specific health disorders or affected bodily systems overall (i.e., regardless of diet), within these 2,054 dogs, were assessed as: gastrointestinal (e.g., diarrhoea, vomiting), skin/coat, musculoskeletal (muscle or bone), ears, mobility problems, dental/oral (teeth/mouth), anal glands, body weight, eyes and cancer/tumours (Table 18). Several previous studies have provided similar results. Among 22,333 UK dogs in 2016, the most prevalent disorder groups were dental, skin, enteropathy and musculoskeletal. When considered individually, the most common disorders were periodontal disease, otitis externa, obesity, overgrown nails and anal sac impaction [26]. An earlier 2009–2013 study of 3,884 English dogs identified the most prevalent disorders as otitis externa, periodontal disease, anal sac impaction, overgrown nails and degenerative joint disease [25]. Analyses of pet insurance records in Sweden indicated skin and gastrointestinal disorders were among the most prevalent [22, 23]. And a telephone survey indicated that the most common disorders in US dogs were musculoskeletal, dental, and gastrointestinal tract or hepatic disease [24].

These results from our 2,054 dogs were broadly consistent with these previous studies, although disorders that were lower in the ‘top 10’ rankings in our sample, seemed to include dental/oral and obesity problems. These differences were even more noteworthy, when considering that our sample included significantly more desexed animals (78% vs 45%) than reported by O’Neill and colleagues [26], yet desexed animals are at greater risk of obesity and dental disorders [26]. Dental disease and obesity are poorly recognized by pet guardians, which may have contributed to this, although we sought to minimise such impacts by relying on reported opinions of veterinarians. Slight differences between our results and those reported in previous studies may also be attributable to the changing prevalence of certain diseases over time, and to differing answer options provided to survey respondents. For example, we did not provide ‘overgrown nails’ as an answer option, although participants had the option to identify musculoskeletal, mobility, or ‘other’ problems, with free text responses allowed for the latter.

When considering these 22 specific disorders individually, very small numbers of affected dogs fed vegan diets in particular (S1 Table), meant that differences compared with other dietary groups, were often not statistically significant. In a small number of cases however, statistically significant differences in disorder prevalence were detectable (S3 Table). Despite the limited generalisability of small numbers, results within our sample were nevertheless interesting in some other cases. The probabilities of suffering from a disorder respectively appeared highest in conventional meat-based dogs (for 11 disorders), raw meat-based dogs (for eight disorders), and vegan dogs (for three disorders) (Fig 10). In some cases, observed differences appeared marked. The dogs in our sample fed vegan diets appeared to have around half the risk of those fed conventional meat diets, of suffering from gastrointestinal disorders (e.g., diarrhoea, vomiting), musculoskeletal (muscle or bone) disorders, ear disorders, anal gland problems, body weight problems, eye problems, behavioural problems, and epilepsy. In all but two of these (body weight and behavioural problems), risks also appeared less than for dogs fed raw meat diets, and sometimes by large margins. Dogs fed vegan diets also appeared to have substantially lowered risks of allergies, compared to either of the other two dietary groups (Fig 10, S2 Table). Some of these differences were particularly noteworthy, given that dogs fed vegan diets were more likely to be neutered, and neutering normally increases risks of obesity, musculoskeletal and behavioural problems [26]. And yet, the dogs in our sample fed vegan diets appeared less likely to suffer from these disorders.

Dogs in our sample fed raw meat diets also appeared to have around half the risk of those fed conventional meat diets, of suffering from gastrointestinal disorders (e.g., diarrhoea, vomiting), body weight problems, hormonal problems (e.g., diabetes, hyper-/hypothyroidism, Addison’s disease, Cushing’s disease), lower urinary tract problems, internal parasites and liver problems. Dogs fed raw meat diets appeared to have substantially lowered risks of behavioural problems, compared to those in either of the other two dietary groups (S2 Table, Fig 10).

The dogs in our sample fed conventional meat diets appeared to have lowered risks of ‘other medical’ problems, injuries and respiratory tract (airways/lungs) problems, than dogs fed raw meat diets, although not in comparison to dogs fed vegan diets. Dogs fed conventional meat diets appeared to have lowered risks of kidney disease when compared to either of the other two dietary groups (S2 Table, Fig 10).

Some of these results match current understanding that some of these disorders may be related. With respect to body weight problems, 85% of affected dogs were reportedly overweight, and such dogs are more likely to experience musculoskeletal problems [36] (p. 783). Dogs who suffer from allergies are more likely to experience skin/coat and ear disorders, all of which appeared less prevalent in dogs fed vegan diets [36] (p. 525).

In some cases, dietary aetiological explanations may exist. Diet is an important allergen source in dogs, and vegan diets lack animal-sourced allergens, such as beef, chicken, fish, pork and lamb [36] (p. 526). In other cases, no immediately obvious aetiological explanation is available, such as apparently increased risks of internal parasites in dogs fed vegan diets, or apparently decreased risks of behavioural disorders in dogs fed raw meat diets. However, vegan pet guardians also appear more likely to feed vegan diets [35]. The vegan lifestyle adhered to by such guardians commonly involves a commitment to minimising harm to living creatures, and it is possible some vegan guardians consider internal parasites to be living creatures deserving of consideration, reducing their use of anthelmintics (de-wormers). It also appears true that certain appetitive behaviours are increased in dogs fed raw meat diets, compared to those fed a conventional diet [21]. Perhaps rates of behavioural disorders could also be affected, although we are not aware of studies assessing this.

#### Consistency with prior studies of vegan dogs

The apparently decreased rates of certain specific disorders in vegan dogs observed in our sample, concur with the results of Semp’s 2014 study [20]. Her questionnaire to 174 vegan dog and 59 vegan cat guardians resulted in 38 reports of healthier and shinier coats after transitioning to vegan diets, and 16 guardians described improved odours of their pets. Some dermatological problems reportedly resolved. As noted, the dogs in our sample fed vegan diets had markedly lowered rates of allergies, compared to either of the other two dietary groups (Fig 10), and in dogs, allergies often manifest as skin conditions [36] (pp. 525–526). And indeed, within our study sample, the probabilities of a dog suffering from a skin/coat condition were 7% in dogs fed conventional meat, 8% in those fed raw meat, and 6% in those fed vegan diets (S2 Table).

Some of Semp’s respondents also noted improved stool consistency. Our results indicated that dogs fed vegan diets also had significantly lowered rates of gastrointestinal problems. Within our study sample, probabilities of dogs suffering from gastrointestinal problems were 11% for dogs fed conventional meat, 6% for those fed raw meat, and 5% for those fed vegan diets (S2 Table).

### Study limitations

When reporting diets fed, guardians were asked to “consider the main ingredients within your pet’s normal diet”. These diets were usually not fed exclusively. Of the 2,536 dogs in the three main diet groups, 76% received a variety of treats at least once daily, and 37% were also regularly offered dietary supplements. Accordingly, our results indicate health outcomes when dogs are fed the three main diet types within normal households, with normal feeding regimes, rather than when dogs are exclusively fed each of the three main diet types, as might occur within a controlled study in a research institute.

Additionally, our study relied on both quantitative information and opinions provided by guardians. The most reliable medical studies are large-scale, prospective studies, that utilise relatively objective assessments of unambiguous data. Veterinary clinical examinations, and veterinary assessments of animal health status, would normally be more reliable than guardian opinions alone, and laboratory results of physiological parameters such as blood and urine tests can provide particularly objective data. However, when large animal numbers are involved, as is necessary for statistical validity of results, such studies become expensive. Unfortunately, such studies were well in excess of our limited research budget.

Accordingly, we were forced to rely on other health indicators. One of these was the answers of guardians (82% of who did not work in the veterinary or pet industries), about health indicators relating to their dogs. We acknowledge that reliance on guardians limits the reliability of results, for example, due to lapses in memory. Our guardians most at risk of this, were those 5% (119/2536) whose animals subsequently progressed onto a therapeutic diet, after initial maintenance on one of the three main diets investigated (Table 7). These guardians were asked to “answer all questions about your animal and their diet, using the 12 months prior to starting their therapeutic or prescription (i.e., medical) diet.” Hence, these guardians were asked to recall details more historical in nature. However, these key instructions were highlighted within the survey, and respondents were also instructed, “If you cannot recall details, please provide your best estimates, or answer ’unsure’ etc. as appropriate.”

Another source of potential error, when relying on guardian answers, is unconscious bias. This could occur if a guardian using a conventional or unconventional pet diet expected a better health outcome as a result, and if this expectation exerted an unconscious effect on their answers about pet health indicators. Our study included more vegans than reported in some other studies [53]. It is conceivable that vegans, or respondents following other dietary groups, such as omnivores, might have had greater subconscious expectations of good health, when animals were fed diets similar to their own. We acknowledge such possible unconscious bias effects cannot be fully eliminated, but to minimise their effects on reported results, we ensured that survey questions asking about animal health were positioned prior to questions about animal diets. This minimises chances that answers might be affected by prior answers about dietary choices, e.g., if a guardian reporting use of an unconventional diet, subsequently became more likely to consciously or unconsciously under-report health problems. Additionally, by careful wording choice, no bias for or against any particular diet was implied within survey advertising materials, or within the survey questions or explanatory text. We do not consider that any remaining unconscious bias effects would be appreciably greater in one dietary group than another; hence consider that their effect on our results was probably minimal, overall.

Despite such steps, reliance on guardian-reported answers is vulnerable to error. We sought to minimise the impact of this unavoidable limitation, by also asking guardians to additionally report the assessment of their veterinarians, concerning their animals’ health. To increase the reliability of such reported veterinary assessments, we included only those guardians whose animals had seen a veterinarian at least once in the previous year, and who were certain of their veterinarian’s assessment. Responses from those who were uncertain, were excluded. And as mentioned, guardians were also given the opportunity to report their own opinion. It was expected the knowledge they would be able to provide their own opinion, if they disagreed with their veterinarian, would encourage them to more accurately report the assessments of their veterinarian. However, we ensured that the analysis of specific health disorders relied on reported veterinary assessments alone, rather than on guardian opinions.

We also asked about several more objective general health indicators, including the frequency of veterinary visits, and the use of any medications, within the previous year, as well as progression onto a therapeutic diet, after being initially fed a conventional meat, raw meat or vegan diet for at least one year. While we accept that a small proportion of these reported data and assessments may have been incorrect, we do not consider it plausible that a significant proportion of them were incorrect.

Our survey was made available from May–December 2020, during the global coronavirus (COVID-19) pandemic. Subsequent lockdowns may have decreased the frequency of veterinary visits in some regions, and potentially, the use of medications or therapeutic diets prescribed by veterinarians. For example, 71% of respondents stated they were from the UK, and in 2020, UK lockdowns occurred during all or part of March, April, July, and September to December [54]. The implementation of remote veterinary consultations and prescribing in many regions may have partly mitigated this effect. Nevertheless, we acknowledge this may have lowered the frequency of some health indicators such as the number of veterinary visits, and medication or therapeutic diet use, to a degree. However, because these were generally indicative of a possible health problem, decreased rates of these would have made our results more conservative overall. We also know of no reason why any one dietary group would be more affected, than any other, in these respects.

We also acknowledge that our respondents were not fully representative of the dog-owning population. Those who lacked easy internet access would have been less likely or unable to complete this internet-based survey. And although most ages were well represented, men were not, representing only 7% of respondents. Most of our participants were also located in the UK (71%) or Europe (15%). However, we do not consider that these anomalies would have significantly affected reported data or opinions concerning the health status of these animals.

Finally, although our participant numbers were sufficient to draw conclusions concerning the overall health of dogs maintained on the three main diets, numbers affected by certain medical disorders may have been insufficient to detect statistically significant differences in risks between diet groups.

### Recommendations for safeguarding health

Within this sample of 2,536 dogs fed three main diets, the reported data and opinions of guardians indicated that dogs that were least healthy when fed conventional meat diets. The health outcomes appeared slightly better for those fed raw meat, than vegan diets. However, the former group enjoyed the health protective effect of being significantly younger, and there were other non-health related factors that may have improved the apparent general health indicators of dogs fed raw meat diets, in three out of seven cases. Accordingly, it is unclear from our study which of these two diets would produce better health outcomes, if these confounding factors were eliminated.

Additionally, all dietary choices may include certain hazards. Those feeding unconventional diets should take special care to ensure their diets are nutritionally complete and reasonably balanced, and appropriate for life stage (e.g., young, old) and physiological status (e.g., pregnant, heavily exercising). Several studies of vegan or vegetarian diets [20, 55, 56], as well as conventional meat diets [57], have demonstrated that some diets in all of these groups have previously been formulated with nutritional deficiencies. Consumers should be encouraged to check labelling claims of nutritional adequacy, and to ask manufacturers what steps they take, and what evidence they can provide, to ensure nutritional soundness and consistency of their diets [17].

Raw meat diets have also been found to have nutritional deficiencies, such as calcium/phosphorous imbalances, and specific vitamin deficiencies [42, 47]. There are also case reports of clinical nutritional disease associated with raw feeding [46, 48]. Additionally, a sizeable body of evidence has indicated that raw meat diets are associated with increased risks of bacterial pathogens, as well as non-bacterial pathogens and zoonoses, with both dogs and their human guardians at increased risk [43–45, 49–51]. For these reasons, raw meat diets are not commonly recommended by veterinarians, and are not recommended by us. Special care should be exercised with respect to food hygiene, by those preparing raw meat diets.

In summary, when jointly considering health outcomes and dietary hazards, our results and those of other studies indicate that the healthiest and least hazardous dietary choices for dogs, are nutritionally sound vegan diets.

### Suggestions for further research

Large-scale cross-sectional, or ideally, longitudinal studies of dogs maintained on different diets, utilising more objective data, such as results of veterinary clinical examinations, veterinary medical histories, and laboratory data, should yield results of greater reliability, if sufficient research funding could be sourced. Whether utilising such an improved research design, or an internet survey, significantly larger numbers might also allow detection of statistically significant differences in risks of specific veterinary medical disorders, between dietary groups. Health consequences within smaller dietary groups, such as vegetarian animals, and of new diets as these become available, could also be investigated. Finally, larger numbers might also allow controlling for possible effects on specific disorders, of factors such as breed, age, sex, neutering status, body condition and weight, exercise levels, seasonality or social factors. This could require limiting to specific groups of interest, rather than dogs in general as in this study, to ensure sample sizes were sufficient to allow detection of statistically significant differences between groups.

## Conclusions

Vegan diets are among a range of alternative diets being formulated to address increasing concerns of consumers about traditional pet foods, such as their ecological ‘pawprint’, perceived lack of ‘naturalness’, health concerns, or impacts on ‘food’ animals used to formulate such diets [8, 9, 35]. Critics have asserted, albeit without evidence, that biological and practical challenges in formulating nutritionally adequate canine vegan diets mean their use should not be recommended [13, 58].

By 2021, no large-scale study of dogs had been published, describing how health outcomes vary between dogs maintained on vegan or meat-based diets. Our study of 2,639 dogs and their guardians is among the first such studies. Among 2,596 respondents who played some role in pet diet decision-making, pet health was one of the most important factors considered.

In total, 2,536 respondents provided information, each relating to a single dog who had been fed a primarily conventional meat (1,370 = 54%), raw meat (830 = 33%) or vegan (336 = 13%) diet for at least one year. Information was provided about seven general health indicators, and 22 specific disorders. Considering all seven general indicators of health, dogs fed conventional meat appeared less healthy than either of the other two dietary groups. They had poorer health indicators in nearly all cases. Considering dogs fed raw meat or vegan diets, the former group had marginally better health indicators overall. However, there was a statistically significant, medium-sized difference in ages, with dogs fed raw meat diets being younger on average. This can provide health protective effects. Other non-health related factors may also have improved the apparent health outcomes of dogs fed raw meat, for three of seven general health indicators. Additionally, a significant body of studies have indicated that raw meat diets commonly include significant dietary hazards, particularly nutritional deficiencies or imbalances, and pathogens. When considering these 22 specific disorders individually, different prevalence levels were apparent between the dietary groups. However, very small numbers of affected dogs fed vegan diets, may have prevented the detection of statistically significant differences in some cases.

Accordingly, when considering health outcomes in conjunction with dietary hazards, the pooled evidence to date from our study, and others in this field, indicates that the healthiest and least hazardous dietary choices for dogs, among conventional, raw meat and vegan diets, are nutritionally sound vegan diets. Regardless of ingredients used, diets should always be formulated to be nutritionally complete and balanced, without which adverse health effects may eventually be expected to occur.

## Supporting information

S1 Table 1,477 cases of 22 specific disorders or affected bodily systems, in 931 dogs fed three main diets, based on reported assessments of veterinarians. (DOCX)

S2 Table Prevalence of 22 specific disorders or affected bodily systems in 2,054 dogs fed three main diets, based on reported assessments of veterinarians. (DOCX)

S3 Table Differences in the likelihood of 22 specific disorders or bodily system effects occurring among 2,054 dogs fed three main diets, based on reported assessments of veterinarians. (DOCX)
