# Correction: Vegan versus meat-based dog food: Guardian-reported indicators of health

**DOI:** 10.1371/journal.pone.0291214

**Published:** 2023-08-31

**Authors:** Andrew Knight, Eason Huang, Nicholas Rai, Hazel Brown

The Competing Interests statement is incorrect. Information about the study’s sponsor is already provided in the Funding statement but is not included in the Competing Interests statement. The correct Competing Interests statement is as follows: This research and its publication open access was funded by food awareness organisation ProVeg International (https://proveg.com). AK received this award ID: Oct2019-0000000286. This does not alter our adherence to PLOS ONE policies on sharing data and materials.

[1]In addition, the Data Availability statement is incorrect. The correct Data Availability statement is as follows: The data analysed are accessible at https://osf.io/nbepu. [2].
