# Supplementary material for: Vegan versus meat-based dog food: Guardian-reported indicators of health
Source: PLoS One. 2022 Apr 13;17(4):e0265662. doi: 10.1371/journal.pone.0265662 (PMC9007375; doi:10.1371/journal.pone.0265662)
Supplement: S1 Table — (DOCX) [file pone.0265662.s001.docx]

# **S1 Table. 1,477 cases of 22 specific disorders or affected bodily systems, in 931 dogs fed three main diets, based on reported assessments of veterinarians.**

| **Disorders (22)** | **Conventional meat** | **Raw meat** | **Vegan** | **Total** |
| --- | --- | --- | --- | --- |
| Allergy | 13 | 7 |  | 20 |
| Anal glands | 69 | 22 | 9 | 100 |
| Behavioural | 43 | 3 | 4 | 50 |
| Body weight | 61 | 12 | 7 | 80 |
| Cancer/tumours | 25 | 18 | 7 | 50 |
| Dental/oral (teeth/mouth) | 75 | 23 | 12 | 110 |
| Ears | 85 | 48 | 9 | 142 |
| Epilepsy | 11 | 5 | 1 | 17 |
| Eyes | 37 | 17 | 4 | 58 |
| Gastrointestinal (e.g., diarrhoea, vomiting) | 129 | 35 | 15 | 179 |
| Heart | 28 | 8 | 9 | 45 |
| Hormonal (e.g., diabetes, hyper-/hypothyroidism, Addison’s, Cushing’s) | 21 | 6 | 4 | 31 |
| Injury | 13 | 12 | 2 | 27 |
| Internal parasites | 7 | 2 | 9 | 18 |
| Kidney | 7 | 7 | 3 | 17 |
| Liver | 9 | 3 | 3 | 15 |
| Lower urinary tract | 18 | 5 | 4 | 27 |
| Mobility | 90 | 29 | 16 | 135 |
| Other medical | 18 | 18 | 4 | 40 |
| Other musculoskeletal (muscle or bone) disease | 92 | 41 | 12 | 145 |
| Respiratory tract (airways/lungs) | 11 | 10 | 3 | 24 |
| Skin/coat | 85 | 46 | 16 | 147 |
| **Total** | **947** | **377** | **153** | **1477** |
