# Supplementary material for: Vegan versus meat-based dog food: Guardian-reported indicators of health
Source: PLoS One. 2022 Apr 13;17(4):e0265662. doi: 10.1371/journal.pone.0265662 (PMC9007375; doi:10.1371/journal.pone.0265662)
Supplement: S2 Table — (DOCX) [file pone.0265662.s002.docx]

# **S2 Table. Prevalence of 22 specific disorders or affected bodily systems in 2,054 dogs fed three main diets, based on reported assessments of veterinarians.**

| **Rank** | **Disorders (22)** | **Conventional meat** | **Raw meat** | **Vegan** | **Overall** |
| --- | --- | --- | --- | --- | --- |
| 1 | Gastrointestinal (e.g., diarrhoea, vomiting) | 11% | 6% | 5% | 9% |
| 2 | Skin/coat | 7% | 8% | 6% | 7% |
| 3 | Other musculoskeletal (muscle or bone) disease | 8% | 7% | 4% | 7% |
| 4 | Ears | 7% | 8% | 3% | 7% |
| 5 | Mobility | 8% | 5% | 6% | 7% |
| 6 | Dental/oral (teeth/mouth) | 6% | 4% | 4% | 5% |
| 7 | Anal glands | 6% | 4% | 3% | 5% |
| 8 | Body weight | 5% | 2% | 3% | 4% |
| 9 | Eyes | 3% | 3% | 1% | 3% |
| 10 | Cancer/tumours | 2% | 3% | 3% | 2% |
| 11 | Behavioural | 4% | 1% | 1% | 2% |
| 12 | Heart | 2% | 1% | 3% | 2% |
| 13 | Other medical | 2% | 3% | 1% | 2% |
| 14 | Hormonal (e.g., diabetes, hyper-/hypothyroidism, Addison’s, Cushing’s) | 2% | 1% | 1% | 2% |
| 15 | Lower urinary tract | 2% | 1% | 1% | 1% |
| 16 | Injury | 1% | 2% | 1% | 1% |
| 17 | Respiratory tract (airways/lungs) | 1% | 2% | 1% | 1% |
| 18 | Allergy | 1% | 1% | 0% | 1% |
| 19 | Internal parasites | 1% | 0% | 3% | 1% |
| 20 | Kidney | 1% | 1% | 1% | 1% |
| 21 | Epilepsy | 1% | 1% | 0% | 1% |
| 22 | Liver | 1% | 1% | 1% | 1% |

**Note:** Ranking is based on overall prevalence of disorders (combining all diets), using exact numbers. In the table, percentages have been rounded to whole numbers.
