# Supplementary material for: Vegan versus meat-based dog food: Guardian-reported indicators of health
Source: PLoS One. 2022 Apr 13;17(4):e0265662. doi: 10.1371/journal.pone.0265662 (PMC9007375; doi:10.1371/journal.pone.0265662)
Supplement: S3 Table — (DOCX) [file pone.0265662.s003.docx]

# **S3 Table. Differences in the likelihood of 22 specific disorders or bodily system effects occurring among 2,054 dogs fed three main diets, based on reported assessments of veterinarians.**

|  |  | **Conventional - Raw meat** | | **Conventional - Vegan** | | **Raw meat - Vegan** | |  |
| --- | --- | --- | --- | --- | --- | --- | --- | --- |
|  |  |  |  |  |  |  |  |  |
| **Rank** | **Disorders (22)** | **Odds ratio** | **P-Value** | **Odds ratio** | **P-Value** | **Odds ratio** | **P-Value** |  |
| 1 | Gastrointestinal (e.g., diarrhoea, vomiting) | **0.508** | **0.001** | **0.463** | **0.006** | 0.911 | 0.768 |  |
| 2 | Skin/coat | 1.076 | 0.699 | 0.783 | 0.384 | 0.727 | 0.288 |  |
| 3 | Other musculoskeletal (muscle or bone) disease | 0.873 | 0.485 | **0.531** | **0.044** | 0.608 | 0.140 |  |
| 4 | Ears | 1.127 | 0.524 | **0.429** | **0.018** | **0.381** | **0.009** |  |
| 5 | Mobility | **0.619** | **0.029** | 0.736 | 0.274 | 1.190 | 0.588 |  |
| 6 | Dental/oral (teeth/mouth) | **0.591** | **0.031** | 0.662 | 0.194 | 1.120 | 0.756 |  |
| 7 | Anal glands | 0.617 | 0.053 | 0.536 | 0.084 | 0.870 | 0.729 |  |
| 8 | Body weight | **0.377** | **0.002** | 0.472 | 0.063 | 1.253 | 0.640 |  |
| 9 | Eyes | 0.906 | 0.741 | 0.449 | 0.131 | 0.495 | 0.210 |  |
| 10 | Cancer/tumours | 1.438 | 0.247 | 1.188 | 0.691 | 0.826 | 0.673 |  |
| 11 | Behavioural | **0.134** | **0.001** | 0.384 | 0.070 | 2.875 | 0.169 |  |
| 12 | Heart | 0.559 | 0.150 | 1.370 | 0.418 | 2.450 | 0.068 |  |
| 13 | Other medical | **2.009** | **0.039** | 0.938 | 0.909 | 0.467 | 0.172 |  |
| 14 | Hormonal (e.g., diabetes, hyper-/hypothyroidism, Addison’s, Cushing’s) | 0.561 | 0.214 | 0.802 | 0.688 | 1.430 | 0.582 |  |
| 15 | Lower urinary tract | 0.546 | 0.233 | 0.938 | 0.909 | 1.719 | 0.422 |  |
| 16 | Injury | 1.843 | 0.130 | 0.648 | 0.569 | 0.351 | 0.173 |  |
| 17 | Respiratory tract (airways/lungs) | 1.812 | 0.177 | 0.769 | 0.734 | 0.425 | 0.271 |  |
| 18 | Allergy | 1.066 | 0.892 | 0.155 | 0.195 | 0.141 | 0.180 |  |
| 19 | Internal parasites | 0.564 | 0.476 | **5.581** | **0.001** | **9.900** | **0.004** |  |
| 20 | Kidney | 1.990 | 0.200 | 1.820 | 0.388 | 0.915 | 0.898 |  |
| 21 | Epilepsy | 0.898 | 0.843 | 0.382 | 0.358 | 0.425 | 0.436 |  |
| 22 | Liver | 0.658 | 0.531 | 1.413 | 0.606 | 2.149 | 0.351 |  |

**Notes:** Ranking is based on overall prevalence of disorders (combining all diets). Statistically significant outcomes are highlighted.
